# Characterization of oral and gut microbiome and plasma metabolomics in COVID-19 patients after 1-year follow-up

**DOI:** 10.1186/s40779-022-00387-y

**Published:** 2022-06-17

**Authors:** Guang-Ying Cui, Ben-Chen Rao, Zhao-Hai Zeng, Xue-Mei Wang, Tong Ren, Hai-Yu Wang, Hong Luo, Hong-Yan Ren, Chao Liu, Su-Ying Ding, Jun-Jie Tan, Zhen-Guo Liu, Ya-Wen Zou, Zhi-Gang Ren, Zu-Jiang Yu

**Affiliations:** 1 Department of Infectious Diseases, the First Affiliated Hospital of Zhengzhou University, Zhengzhou, 450052 China; 2 Gene Hospital of Henan Province/Precision Medicine Center, the First Affiliated Hospital of Zhengzhou University, Zhengzhou, 450052 China; 3 Department of Infectious Diseases, Guangshan County People’s Hospital, Guangshan County, Xinyang, 465450 Henan China; 4 Department of Breast Surgery, Affiliated Cancer Hospital of Zhengzhou University, Zhengzhou, 450052 China; 5 Shanghai Mobio Biomedical Technology Co., Ltd, Shanghai, 201111 China; 6 Health Management Center, the First Affiliated Hospital of Zhengzhou University, Zhengzhou, 450052 China

**Keywords:** Coronavirus disease 2019 (COVID-19), Gut microbiome, Oral microbiome, Plasma metabonomics, Convalescents, Individual outcomes, Patient stratification, Predictive, Preventive and personalized medicine (3PM)

## Abstract

**Background:**

Due to the outbreak and rapid spread of coronavirus disease 2019 (COVID-19), more than 160 million patients have become convalescents worldwide to date. Significant alterations have occurred in the gut and oral microbiome and metabonomics of patients with COVID-19. However, it is unknown whether their characteristics return to normal after the 1-year recovery.

**Methods:**

We recruited 35 confirmed patients to provide specimens at discharge and one year later, as well as 160 healthy controls. A total of 497 samples were prospectively collected, including 219 tongue-coating, 129 stool and 149 plasma samples. Tongue-coating and stool samples were subjected to 16S rRNA sequencing, and plasma samples were subjected to untargeted metabolomics testing.

**Results:**

The oral and gut microbiome and metabolomics characteristics of the 1-year convalescents were restored to a large extent but did not completely return to normal. In the recovery process, the microbial diversity gradually increased. Butyric acid-producing microbes and *Bifidobacterium* gradually increased, whereas lipopolysaccharide-producing microbes gradually decreased. In addition, sphingosine-1-phosphate, which is closely related to the inflammatory factor storm of COVID-19, increased significantly during the recovery process. Moreover, the predictive models established based on the microbiome and metabolites of patients at the time of discharge reached high efficacy in predicting their neutralizing antibody levels one year later.

**Conclusions:**

This study is the first to characterize the oral and gut microbiome and metabonomics in 1-year convalescents of COVID-19. The key microbiome and metabolites in the process of recovery were identified, and provided new treatment ideas for accelerating recovery. And the predictive models based on the microbiome and metabolomics afford new insights for predicting the recovery situation which benefited affected individuals and healthcare.

**Supplementary Information:**

The online version contains supplementary material available at 10.1186/s40779-022-00387-y.

## Background

Due to the outbreak and rapid spread of coronavirus disease 2019 (COVID-19), more than 160 million patients have become convalescents worldwide to date [1]. The pathophysiological changes caused by COVID-19 can have long-term effects on the body and cause persistent symptoms [2, 3]. A 6-month follow-up of COVID-19 patients found that fatigue, sleep disturbance and dyspnea were the most common symptoms [4–6]. Therefore, it is very important to pay more attention to the health condition of convalescents in the context of predictive, preventive and personalized medicine (3PM) approach [7, 8].

Alterations in the human microbiome are closely related to various diseases. Our previous study described the characteristics of the oral microbiome of patients with COVID-19. At the genus level, 5 genera were increased, including *Selenomonas* and *Leptotrichia,* while 5 genera were decreased, including *Fusobacterium* and *Porphyromonas*. In addition, we constructed a diagnostic model based on the oral microbiome and achieved good diagnostic performance [9]. Alterations in the gut microbiome took place and reflected the disease severity of COVID-19 [10]. Compared with healthy controls, the fecal microbial diversity of patients with COVID-19 was significantly decreased, which was characterized by enrichment of opportunistic pathogens and exhaustion of beneficial symbiotic bacteria. At the phylum level, *Bacteroides* in COVID-19 patients was significantly increased, while *Actinomycetes* was significantly decreased [9, 11]. Therefore, it is necessary to explore the characteristics and evaluate the recovery situation of the human microbiome for COVID-19 patients after a 1-year recovery.

The pathophysiological changes of many diseases can lead to alterations in human metabolites [12–14]. Researchers identified 941 metabolites in the serum and constructed a great diagnostic model for COVID-19 [15, 16]. Our previous study reported the characteristics of lipid metabolomics in patients with COVID-19. During the treatment process, 122 lipid molecules, including diglyceride (20:1/18:2), were increased, while 47 lipid molecules, including monoglyceride (33:5), were decreased [9]. Thus, studying the characteristics and assessing the recovery situation of the metabolites for COVID-19 patients after a 1-year recovery are of great importance.

In this study, we characterized the oral and gut microbiomes and plasma metabonomics of 35 1-year convalescents. Then, the key microbiome and metabolites during the recovery process were identified. Furthermore, models based on the microbiome and metabolites were constructed to predict the level of neutralizing antibodies in 1-year convalescents.

## Methods

### Study profile

This study was conducted following prospective specimen collection and retrospective blinded evaluation design principles. Ethical approval for this study was granted by the Ethics Committee of the First Affiliated Hospital of Zhengzhou University (2020-KY-055) and Guangshan Country People’s Hospital (2020–001). Written informed consent was collected from each participant.

A total of 35 confirmed patients were recruited and completed a 1-year follow-up after recovery. In addition, 160 healthy controls (HCs) matched with COVID-19 patients in gender and age were enrolled in the Health Management Center, the First Affiliated Hospital of Zhengzhou University. Tongue-coating samples, stool samples and plasma samples were provided by the participants. 16S rRNA MiSeq sequencing was conducted on tongue-coating samples and stool samples, and untargeted metabolomics testing on liquid chromatography-mass spectrometry (LC–MS) analysis was carried out on plasma samples.

### Tongue-coating and stool sample collection and DNA extraction

Each participant provided a fresh tail stool and tongue-coating sample from 7 to 9 am. Tongue-coating and stool samples were collected as described in our previous study [9] (Additional file 1: Supplementary methods). To ensure the quality of the samples, we stored the samples at − 80 °C as soon as possible, and all samples that had been at room temperature for more than 2 h were excluded. The DNA extraction process was as described in our previous study [17] (Additional file 1: Supplementary methods).

### PCR amplification, MiSeq sequencing and data processing

PCR amplification and DNA library construction were conducted based on standard protocols, and sequencing was accomplished on an Illumina MiSeq platform by Shanghai Mobio Biomedical Technology, China [9]. Detailed information on PCR amplification and sequence data processing was uploaded to the Additional file 1: Supplementary methods. The raw Illumina read data for all samples were available through the European Nucleotide Archive at the European Bioinformatics Institute under accession number PRJNA756623.

### Operational taxonomy unit (OTU) clustering and taxonomy annotation

Select all samples with equal numbers for random reads and bin the OTUs by UPARSE pipeline. The identity threshold was set as 0.97. We used RDP classifier version 2.6 [18]; the confidence level was set as 0.5 to annotate sequences [19]. The details of microbial diversity and taxonomic analysis are shown in the Additional file 1: Supplementary methods.

### Identification of the OTU biomarkers and construction of probability of disease (POD)

The Wilcoxon rank-sum test was used to determine the significance (*P* < 0.05), and OTU biomarkers in the gut and oral microbiomes were selected for further analysis. The prediction model was constructed on a random forest model through fivefold cross-validation, and then, we evaluated the probability of disease (POD) index and receiver operating characteristic curve. The details of POD construction were performed as described in our previous study [17] (Additional file 1: Supplementary methods).

### Untargeted metabolomics detection and data analysis

Untargeted metabolomics testing on LC–MS analysis was carried out on all plasma samples. LC–MS analysis was performed using an Orbitrap Elite high-resolution mass spectrometer (Thermo-Finnigan). Details about untargeted metabolomics detection and data analysis are shown in the Additional file 1: Supplementary methods.

### Immunoassay of severe acute respiratory syndrome coronavirus 2 (SARS-CoV-2) neutralizing antibody and IgG

The SARS-CoV-2 neutralizing antibody was detected based on the ELISA method [20]. SARS-CoV-2 IgG assays were conducted by direct chemiluminometric microparticle technology [21]. Details are described in the Additional file 1: Supplementary methods.

### Statistical analysis

Statistical analyses were performed using SPSS v.20.0 for Windows (SPSS, Chicago, Illinois, USA). Continuous variables between two groups were compared by Student’s *t*-test or Wilcoxon rank-sum test. Normally distributed values are presented as mean ± SD (standard deviation), and Nonnormally distributed values are presented as median (interquartile range). Categorical variables between two groups were compared by the *χ*^2^ test or Fisher’s exact test. Differences among three groups were evaluated by one-way analysis of variance. A *P*-value < 0.05 (two-sided) was considered to indicate a significant difference.

## Results

### Study design and flow diagram

**Fig. 1:**
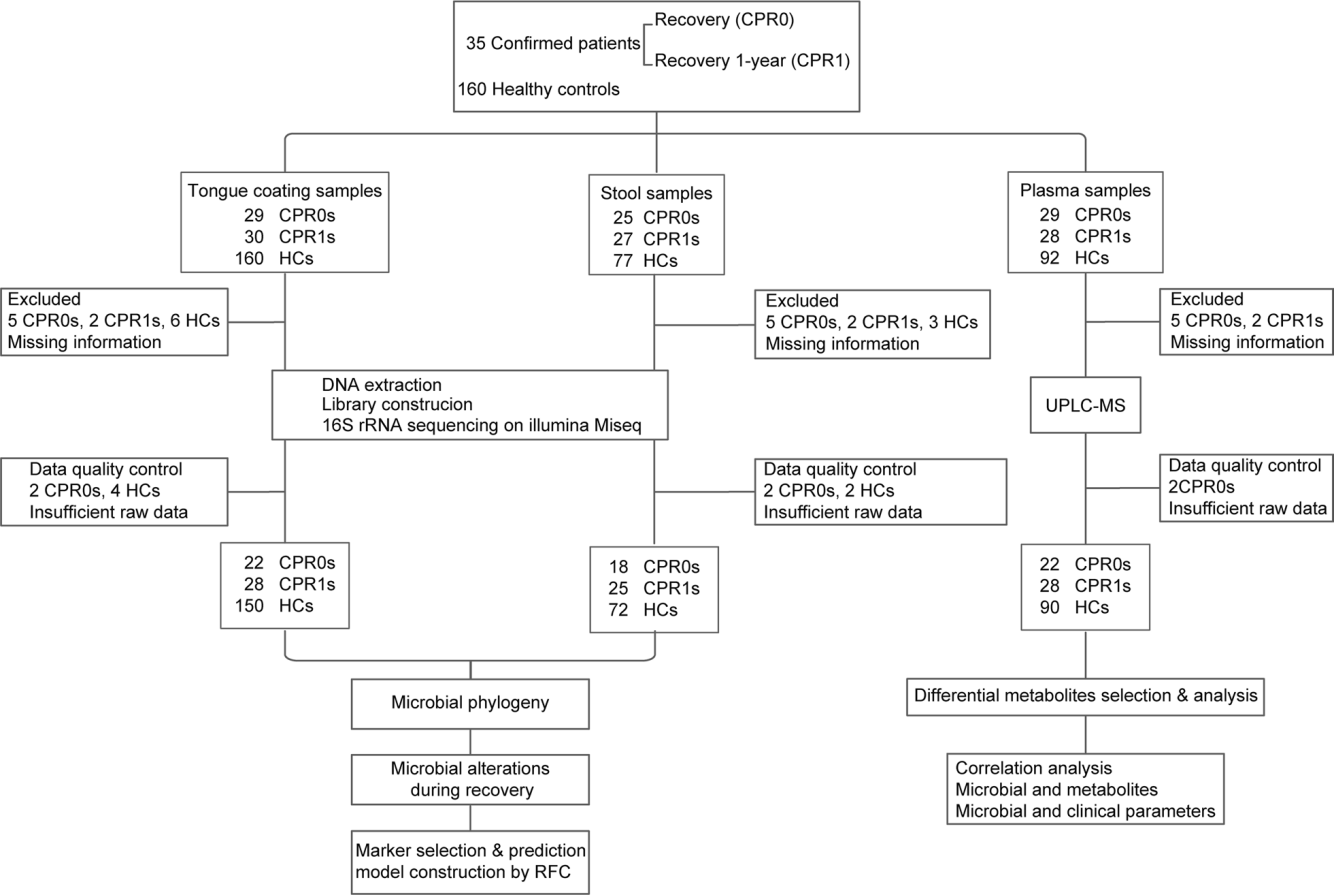
Study design and flow diagram. A total of 35 confirmed patients were recruited and completed a 1-year follow-up after recovery. In addition, 160 healthy controls (HCs) were enrolled. A total of 497 samples were prospectively collected, including 219 tongue-coating samples, 129 stool samples and 149 plasma samples. In addition, 455 samples were included for further analysis after strict inclusion and exclusion criteria. 16S rRNA MiSeq sequencing was conducted on tongue-coating samples and stool samples, and untargeted metabolomics testing on liquid chromatography-mass spectrometry (LC–MS) analysis was carried out on plasma samples. CPR0 confirmed patients recover at discharge, CPR1 confirmed patients recover 1 year, HCs healthy controls, UPLC-MS ultra-performance liquid chromatography-mass spectrometry, RFC random forest model

The confirmed patients provided specimens at the time of recovery discharge (CPR0) and recovered 1 year (CRP1). A total of 497 samples were prospectively collected from participants. After strict exclusion criteria, 455 samples were included for further analysis, including 200 tongue-coating samples, 115 stool samples and 140 plasma samples (Fig. 1). In this study, we described the characteristics of the oral and gut microbiomes and plasma metabolomics of CPR1s. Then, we identified the key microbiome and metabolites during the recovery process of the confirmed patient. Finally, we constructed prediction models based on the microbiome and metabolites to forecast the neutralizing antibodies of CRP1s.

### Characteristics of the participants

The clinical characteristics of CPR1s and HCs are shown in Additional file 2: Table S1. In this study, the gender and age of the two groups of participants were matched. There was no significant difference in routine blood indicators between the two groups of participants. In addition, the liver and kidney function indices of CPR1s were similar to those of HCs. Thus, these clinical indices indicated that the overall health of CPR1 has returned to normal. To clarify the recovery of the oral and gut microbiome and metabolites, we conducted the following study.

### Gradual recovery of the oral microbiome

**Fig. 2:**
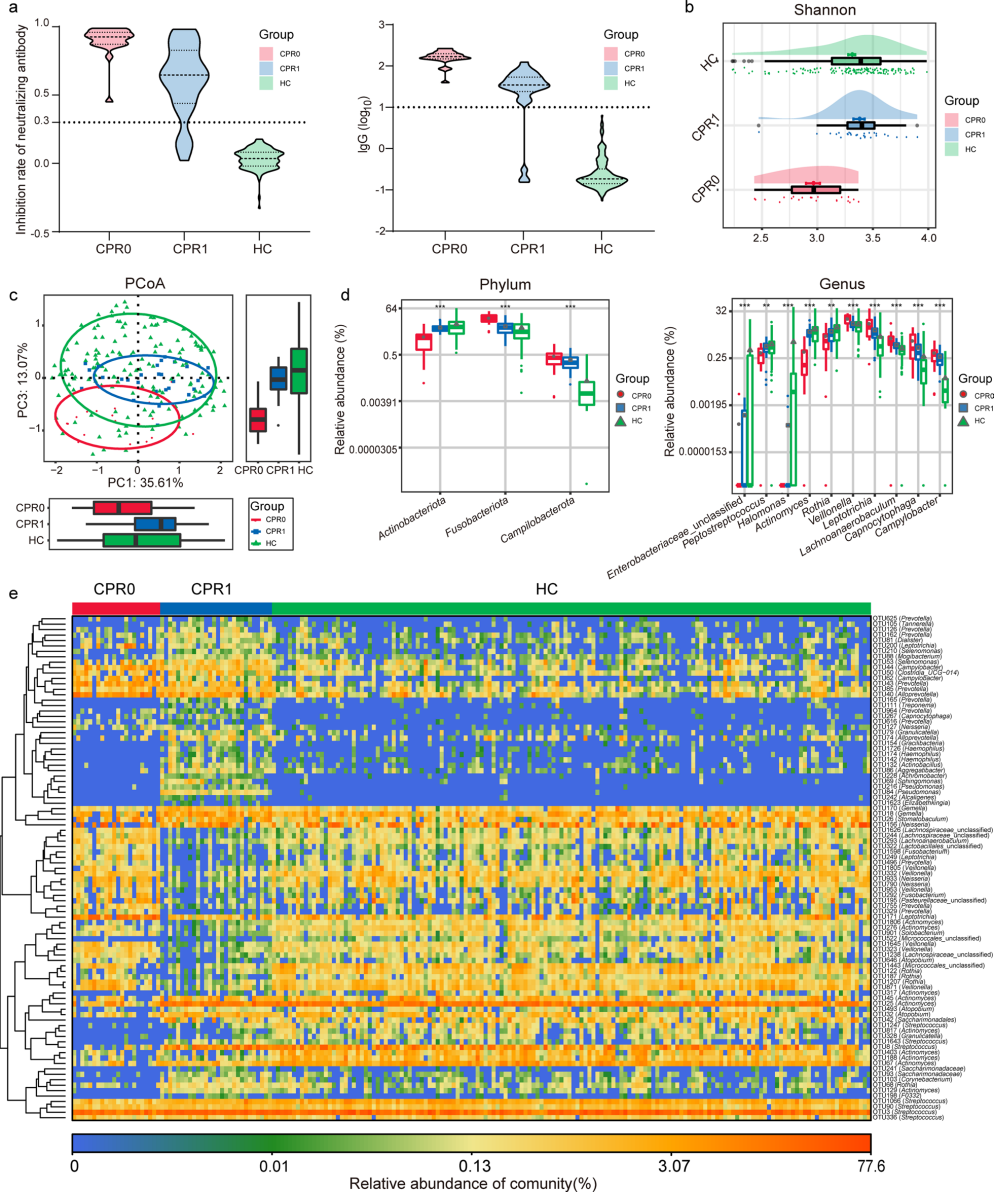
Gradual recovery of the oral microbiome. **a** Neutralizing antibodies and IgG gradually decreased from CPR0 to CPR1 to HC. **b** As estimated by the Shannon index, the oral microbial α diversity gradually increased. **c** The PCoA showed that the compositions and β diversity gradually recovered. **d** Key oral bacteria whose abundance gradually increased or decreased were identified at the phylum and genus levels among three groups. **e** Heatmap displays the gradual recovery of the key OTUs in the oral microbiome. ^**^P < 0.01, ^***^P < 0.001, compared among three groups. CPR0 confirmed patients recover at discharge, CPR1 confirmed patients recover 1 year, HC healthy control, PCoA principal coordinate analysis, OTUs operational taxonomy units

Violin plot graphs showed alterations in neutralizing antibodies and IgG during the recovery process of COVID-19 (Fig. 2a, Additional file 2: Table S2). The antibody level of CPR1s was significantly lower than that of CPR0s; however, it was significantly higher than that of HCs (*P* < 0.05). The oral microbial α diversity was significantly decreased, which was an important characteristic of confirmed patients [9]. Then, the α diversity analysis showed that the α diversity of the oral microbiome gradually increased as COVID-19 recovered (Fig. 2b, Additional file 2: Table S3). The abundance of rare species was significantly increased during the recovery process according to the Chao index and observed OTUs index (*P* < 0.05, Additional file 3: Fig. S1a). The Venn diagram shows the shared and unique oral microbial OTUs between the three groups (Additional file 3: Fig. S1b). In addition, β diversity indicated the process of gradually returning to normal by principal coordinate analysis (PCoA), principal component analysis (PCA) and nonmetric multidimensional scaling (NMDS) analysis (Fig. 2c, Additional file 3: Fig. S1c).

The average composition of the oral microbiome among the three groups was similar (Additional file 2: Table S4; Additional file 3: Fig. S1d). At the genus level, the abundances of *Prevotella*, *Neisseria*, *Veillonella*, *Streptococcus* and *Porphyromonas* accounted for 65% of all species. In addition, linear discriminant analysis effect size (LEfSe) was used to compare the estimated phylotypes of the oral microbiome. The oral microbial characteristics were illustrated at the genus level among the three groups [LDA score (log_10_) > 3] (Additional file 2: Table S5, Additional file 3: Fig. S1e). At the phylum level, in the process of recovery, the abundance of *Actinobacteriota* gradually increased. In contrast, the abundance of *Campilobacterota* and *Fusobacteriota* gradually decreased (*P* < 0.001, Fig. 2d, Additional file 2: Table S6). At the genus level, in the process of recovery, the abundance of unclassified *Enterobacteriaceae*, *Peptostreptococcus*, *Halomonas*, *Actinomyces* and *Rothia* gradually increased. In contrast, the abundances of *Veillonella*, *Leptotrichia*, *Lachnoanaerobaculum*, *Capnocytophaga* and *Campylobacter* gradually decreased (*P* < 0.001, Fig. 2d). The heatmap displayed the gradual recovery of the key OTUs in the oral microbiome (Fig. 2e).

### Noninvasive prediction model for CPR1 neutralizing antibodies based on the oral microbiome

In this study, there were 16 participants in CPR0s and CPR1s in a one-to-one correspondence. We analyzed the oral microbiome of these 16 CPR0s. The 16 CPR0s were divided into confirmed patients who recovered at discharge with higher neutralizing antibodies 1 year later (CPR0-H) and those with lower neutralizing antibodies 1 year later (CPR0-L) according to the cutoff value of 70% of the inhibition rate of neutralizing antibodies. One year later, the neutralizing antibody and IgG levels of CPR0-L were significantly lower than those of CPR0-H (*P* < 0.05, Additional file 2: Table S7; Additional file 3: Fig. S2a). There was no significant difference in the α diversity of the oral microbiome between CPR0-L and CPR0-H (*P* > 0.05, Additional file 2: Table S8, Additional file 3: Fig. S2b). The Venn diagram showed that 434 of 679 OTUs were common to both groups, while 175 OTUs were unique to CPR0-L (Additional file 3: Fig. S2c). The key oral microbiome with significant differences between the two groups was identified at the phylum and genus levels. *Campilobacterota* was significantly decreased in CPR0-H versus CPR0-L at the phylum level, and *Campylobacter* was enriched in CPR0-L at the genus level (*P* < 0.05, Additional file 2: Table S9, Additional file 3: Fig. S2d).

To illustrate the predicted value of the oral microbiome for CPR1 neutralizing antibodies, we constructed a random forest prediction model that could specifically identify whether CPR1 neutralizing antibodies were less than 70%. To identify unique OTUs markers, we conducted fivefold cross-validation on a random forest model between CPR0-H and CPR0-L. The analysis showed that the 9 OTUs markers were selected as the optimal marker set (Additional file 3: Fig. S2e). We defined the POD index as the probability that the neutralizing antibody inhibition rate of CPR1 was less than 70%. The POD index was significantly increased in CPR0-L compared with CPR0-H (*P* < 0.05, Additional file 3: Fig. S2f). The POD index achieved an AUC value of 0.8413 (95% CI 0.6393–1) between CPR0-L and CPR0-H (Additional file 2: Table S10, Additional file 3: Fig. S2g). The AUC curve showed a high prediction efficiency, confirming the ability of the CPR0 oral microbiome to predict the neutralizing antibodies of CPR1s.

### Gradual recovery of the gut microbiome

**Fig. 3:**
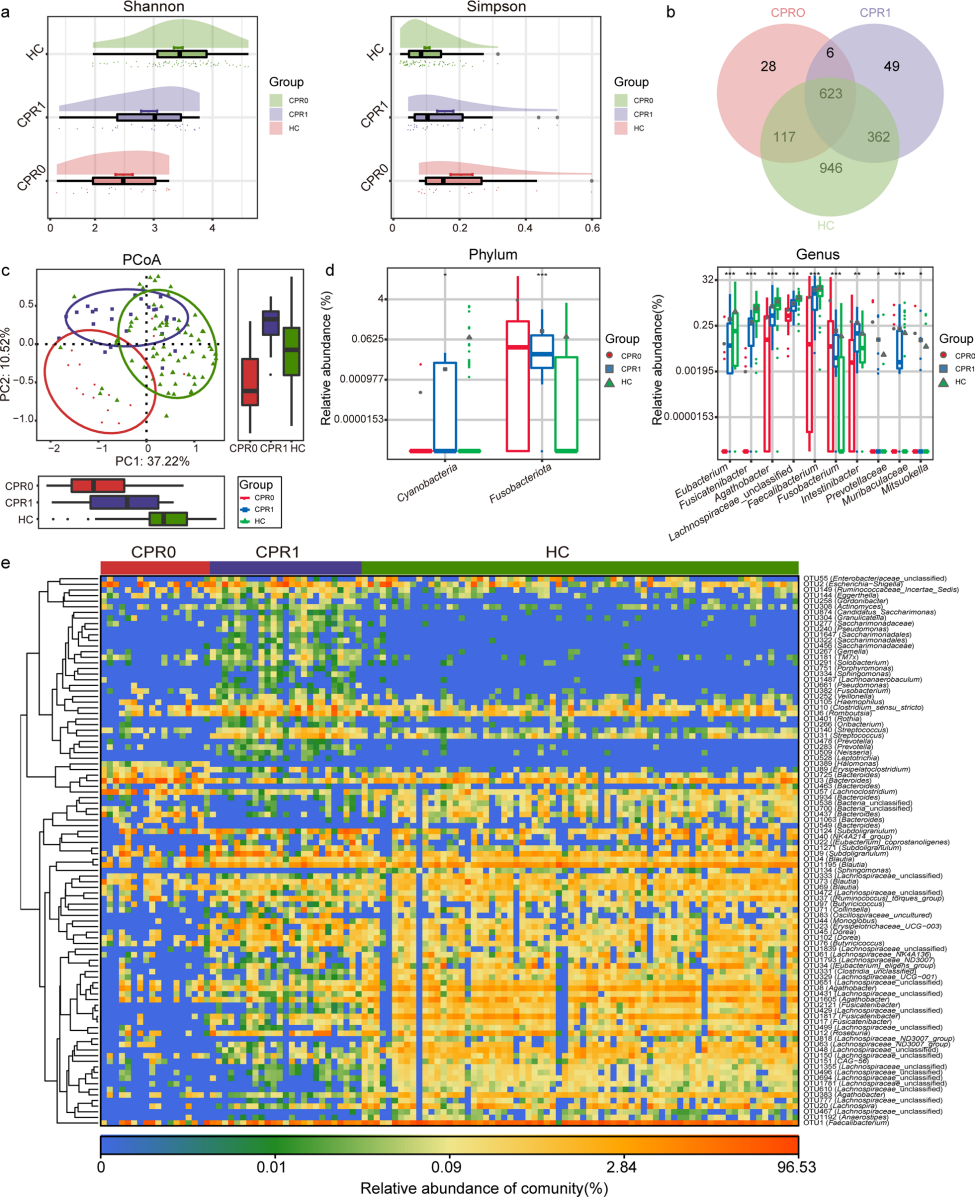
Gradual recovery of the gut microbiome. **a** As estimated by the Shannon index and Simpson index, gut microbial diversity gradually increased during the recovery process. **b** Venn diagram displayed the shared and different OTUs between the three groups. **c** PCoA showed that the compositions and diversity gradually recovered. **d** In the process of recovery, the key gut bacteria whose abundance gradually increased or decreased were identified among three groups. **e** Heatmap displays the gradual recovery of the key OTUs in the gut microbiome. ^*^P < 0.05, ^**^P < 0.01, ^***^P < 0.001, compared among three groups. CPR0 confirmed patients recover at discharge, CPR1 confirmed patients recover 1 year, HC healthy control, PCoA principal coordinate analysis, OTUs operational taxonomy units

The alterations of the neutralizing antibody and IgG during the recovery process of COVID-19 are displayed in Fig. 2a. Then, the Shannon index and Simpson index of the gut microbiome showed that the α diversity of the gut microbiome gradually increased as COVID-19 recovered (*P* < 0.05, Fig. 3a, Additional file 2: Table S11). Furthermore, the Venn diagram, PCoA, PCA and NMDS analysis indicated the process of gradually returning to normal of the gut microbiome (Fig. 3b, c; Additional file 3: Fig. S3a). Compared with the oral microbiome, we could conclude that the gut microbiome takes longer to return to normal.

The average composition of the gut microbiome among the three groups was evaluated, and the domain species of the gut microbial community are shown in Additional file 3: Fig. S3b. The key gut bacteria that changed gradually during the recovery process were identified. The gut microbial characteristics were described at the genus level among the three groups [LDA score (log_10_)] > 4] (Additional file 2: Table S12, Additional file 3: Fig. S3c). At the phylum level, in the process of recovery, the abundance of *Cyanobacteria* gradually increased (*P* < 0.05). In contrast, the abundance of *Fusobacteriota* gradually decreased (*P* < 0.001, Fig. 3d, Additional file 2: Table S13). At the genus level, in the process of recovery, the abundances of *Eubacterium*, *Fusicatenibacter*, *Agathobacter*, unclassified *Lachnospiraceae* and *Faecalibacterium* gradually increased. However, the abundances of *Fusobacterium*, *Intestinibacter*, *Prevotellaceae*, *Muribaculaceae* and *Mitsuokella* gradually decreased (*P* < 0.05, Fig. 3d). The heatmap displayed the alterations of the key OTUs with significant differences in the gut microbiome during recovery (Fig. 3e, Additional file 2: Table S14).

### Noninvasive prediction model for CPR1 neutralizing antibodies based on the gut microbiome

The difference in the diversity index of the gut microbiome between CPR0-L and CPR0-H was not significant (*P* > 0.05, Additional file 2: Table S15, Additional file 3: Fig. S4a). The Venn diagram showed that 348 of 719 OTUs were common to both groups, while 158 OTUs were unique to the CPR0-L group (Additional file 3: Fig. S4b). At the family level, *marinifilaceae* was significantly depleted in CPR0-L vs. CPR0-H (*P* < 0.05, Additional file 2: Table S16, Additional file 3: Fig. S4c). *Butyricimonas,* which was significantly reduced in CPR0-L vs. CPR0-H at the genus level (*P* < 0.05, Additional file 3: Fig. S4d).

We constructed a random forest prediction model that could specifically identify whether CPR1 neutralizing antibodies were less than 70% to illuminate the predicted value of the gut microbiome in CPR1 neutralizing antibodies. A fivefold cross-validation was conducted on a random forest model between CPR0-H and CPR0-L to identify distinct OTUs markers. Then, 6 OTUs markers, OTU902, OTU58, OTU1563, OTU131, OTU187 and OTU130, were selected as the optimal marker set (Additional file 3: Fig. S4e). We calculated the POD index for each sample. The POD index was significantly increased in CPR0-L compared with CPR0-H (*P* < 0.05, Additional file 2: Table S17, Additional file 3: Fig. S4f). The POD index achieved an AUC value of 0.8571 (95% CI 0.5733–1) between CPR0-L and CPR0-H (Additional file 3: Fig. S4g). In conclusion, the prediction model based on the CPR0 gut microbiome achieved a high prediction efficiency in predicting the neutralizing antibodies of CPR1s.

### Gradual recovery of the plasma metabonomics

**Fig. 4:**
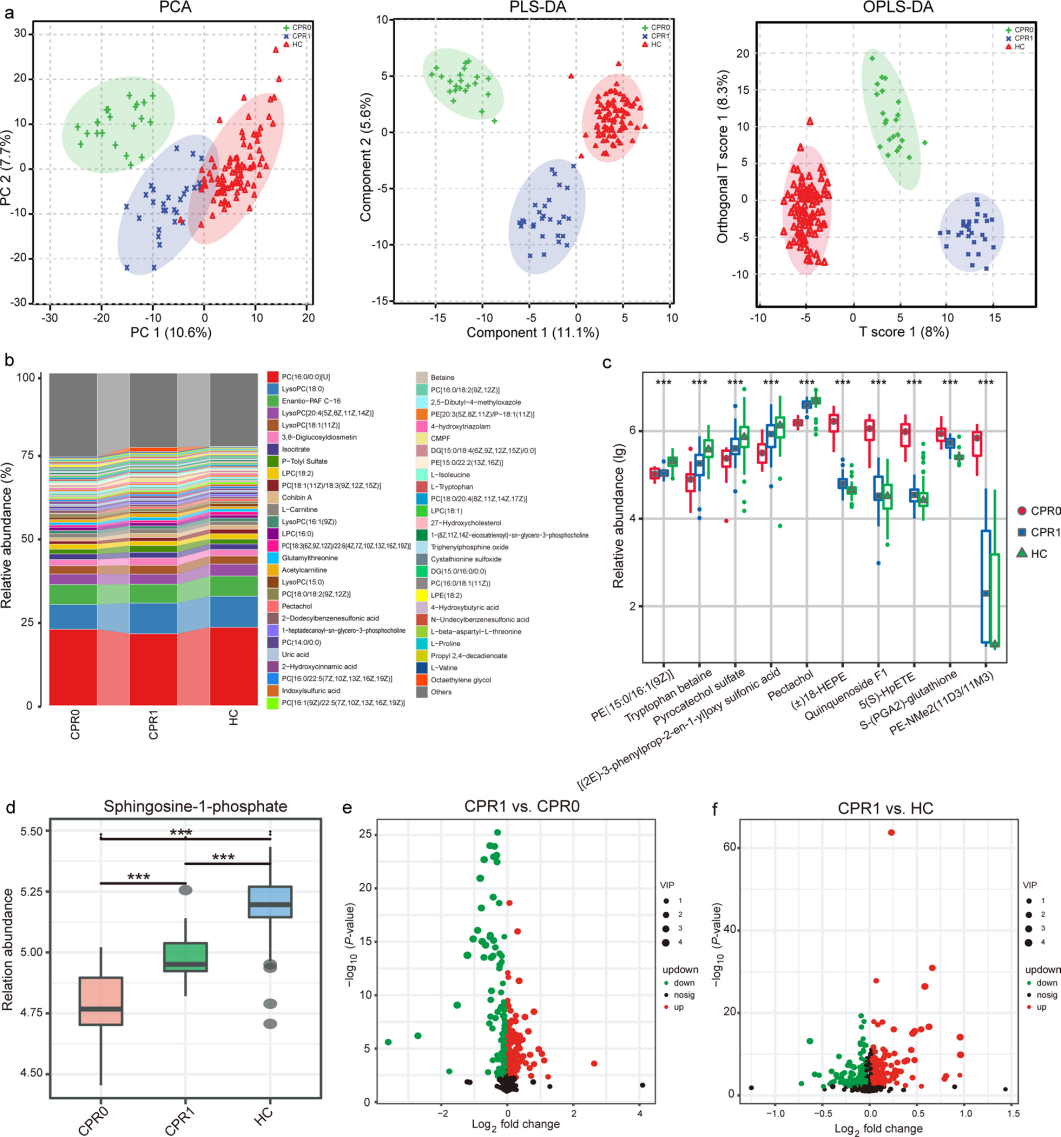
Gradual recovery of the plasma metabonomics.** a** PCA, PLD-DA and OPLS-DA showed the gradual recovery of plasma metabonomics. **b** Average compositions and relative abundance of the metabonomics community among the three groups. **c** In the process of recovery, the key metabolites whose abundance gradually increased or decreased were identified. **d** Sphingosine-1-phosphate increased significantly during the recovery process. **e** Annotated metabolites were analyzed and compared between CPR1 and CPR0. **f** Annotated metabolites were analyzed and compared between CPR1 and HC.^***^P < 0.001, pairwise comparisons among the three groups. CPR0 confirmed patients recover at discharge, CPR1 confirmed patients recover 1 year, HC healthy control, PCA principal component analysis, PLS-DA partial least squares discrimination analysis, OPLS-DA orthogonal partial least squares discrimination analysis

The metabolomics characteristics of COVID-19 patients have undergone significant changes[15]; thus, we continued to explore the metabolomics characteristics of CPR1. Samples from 22 CPR0s, 28 CPR1s and 90 HCs were eventually used for LC–MS untargeted metabonomics tests (Additional file 2: Tables S18-S19). PCA, partial least squares discrimination analysis (PLS-DA) and orthogonal partial least squares discrimination analysis (OPLS-DA) were conducted to compare the metabolites among CPR0, CPR1 and HC. From CPR0 to CPR1 to HC, the metabolites showed a significant trend of gradual recovery (Fig. 4a). The average composition of the plasma metabolites among the three groups is shown in Fig. 4b and Additional file 2: Table S20. PC(16:0/0:0)[U], LysoPC(18:0), Enantio-PAF C-16, LysoPC[20:4(5Z,8Z,11Z,14Z)], LysoPC[18:1(11Z)] and 3,8-Diglucosyldiosmetin were the dominant metabolites, accounting for 40% of the three groups. In the process of recovery, the abundances of PE[15:0/16:1(9Z)], tryptophan betaine, pyrocatechol sulfate, [(2E)-3-phenylprop-2-en-1-yl]oxy sulfonic acid and pectachol gradually increased. However, the abundances of ( ±)18-HEPE, quiquenoside F1, 5(S)-HpETE, S-(PGA2)-glutathione and PE-NMe2(11D3/11M3) gradually decreased (*P* < 0.001, Fig. 4c, Additional file 2: Table S21). Sphingosine-1-phosphate (S1P), which is closely related to the inflammatory factor storm of COVID-19, increased significantly during the recovery process [22](*P* < 0.001, Fig. 4d).

We analyzed the annotated metabolites and found 204 differential metabolites between the CPR1 and CPR0 groups (VIP > 1, *P* < 0.05), among which 89 metabolites, including2,3-dihydro-6-methyl-5-(5-methyl-2-furanyl)-1H-pyrrolizine (VIP = 2.57, fold change = 0.084), were downregulated and 115 metabolites, including leukotriene E3 (VIP = 1.88, fold change = 6.315), were upregulated in CPR1 (Fig. 4e). The differential metabolites between the CPR1 and HC groups are shown in Fig. 4f. Furthermore, we studied the correlation between different metabolites. The correlation between 204 different metabolites from CPR1 and CPR0 is shown (Additional file 2: Table S22, Additional file 3: Fig. S5). In addition, 216 different metabolites were identified between CPR1 and HC, and Additional file 2: Table S23 and Additional file 3: Fig. S6 described the correlation between different metabolites.

### Noninvasive prediction model for CPR1 neutralizing antibodies based on plasma metabolomics

Additionally, we analyzed the difference in metabonomics between CPR0-L and CPR0-H (Additional file 2: Tables S24-S25). Overall, there was no significant difference in the composition between the two groups (Additional file 3: Fig. S7a). The dominant species in CPR0-L and CPR0-H were similar (Additional file 2: Table S26, Additional file 3: Fig. S7b). The abundance of 23 metabolites, including ( ±)18-HEPE and quinquenoside F1, was significantly decreased in CPR0-L. However, the abundance of 11 metabolites, including 4-acetamidobutanoate and LysoPC (17:0), was enriched in CPR0-L (*P* < 0.05, Additional file 2: Table S27, Additional file 3: Fig. S7c). Annotated metabolites were analyzed and compared between CPR0-L and CPR0-H (Additional file 3: Fig. S7d).

To illuminate the predicted value of plasma metabonomics, a random forest predicted model that could specifically identify whether CPR1’ neutralizing antibody was less than 70%. Fivefold cross-validation was conducted on a random forest model between CPR0-H and CPR0-L to determine distinct metabonomics markers. As a result, 22 metabolites, including PC [17:1(9Z)/0:0] and quinquenoside F1, were selected as the optimal marker set (Additional file 3: Fig. S7e). The POD index was calculated for each sample. The POD index was significantly increased in CPR0-L versus CPR0-H (*P* < 0.05, Additional file 2: Table S28, Additional file 3: Fig. S7f). In addition, the POD index achieved an AUC value of 0.8704 (95% CI 0.6675—1) between CPR0-L and CPR0-H (Additional file 3: Fig. S7g). The AUC curve indicated the strong ability of CPR0s’ plasma metabolites to predict CPR1’ neutralizing antibodies.

### Correlation in the gradual recovery process from CPR0 to CPR1 to HC

**Fig. 5:**
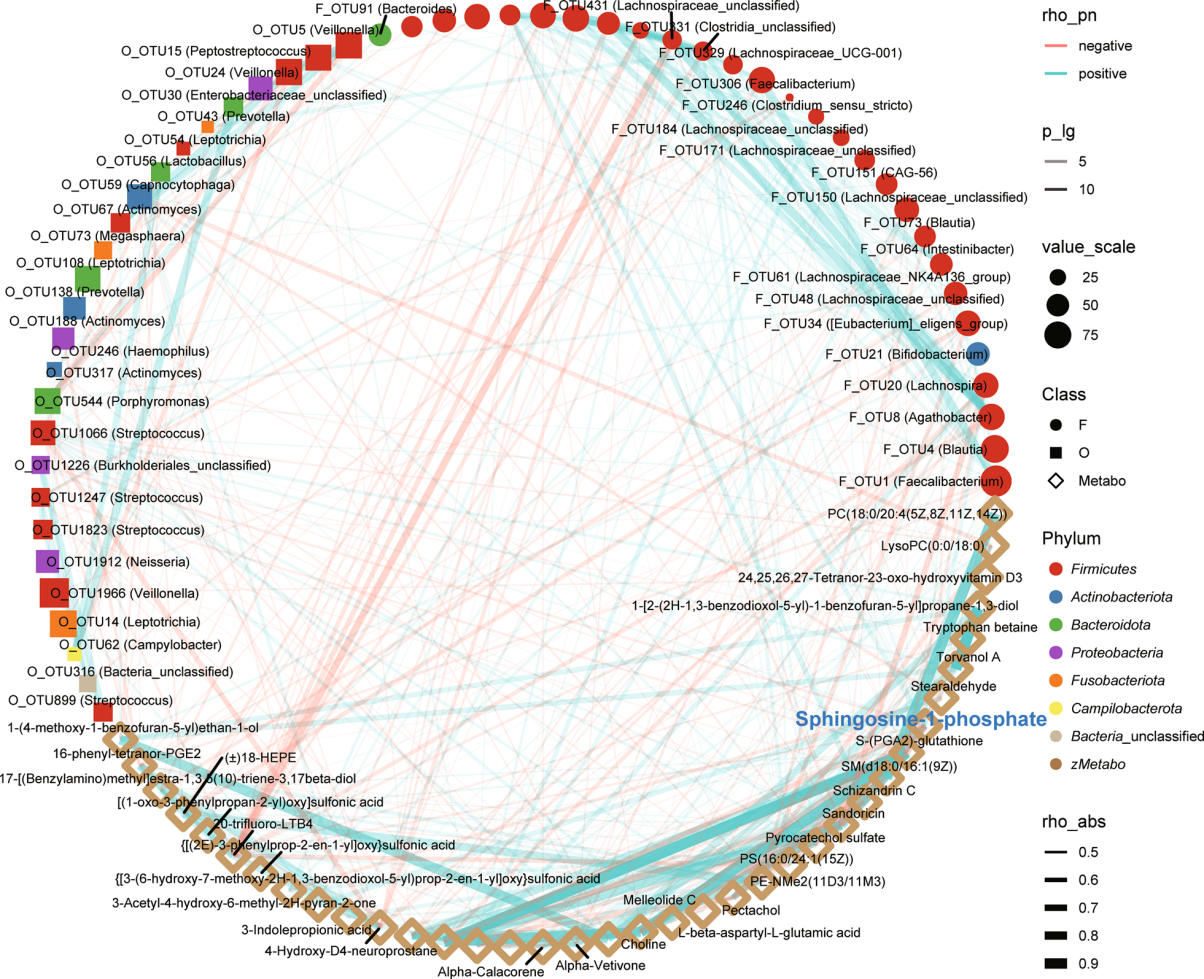
Correlation in the gradual recovery process from CPR0 to CPR1 to HC. The correlations among the oral and gut microbiomes and metabonomics were analyzed in the gradual recovery process from CPR0 to CPR1 to HC. We included microbiomes and metabolites that showed an increasing or decreasing trend during the recovery process for Spearman’s correlation analysis. The results showed correlations among 26 fecal microbial OTUs, 28 oral microbial OTUs and 37 metabolites. Red lines indicate negative correlations, blue lines indicate positive correlations, and the width of the lines represents the strength of the correlation (Spearman). The transparency of the lines represented the negative logarithm of the *P*-value of correlation, translucent lines meant (-lgP) > 5 and opaque lines meant (-lgP) > 10. The size of the points indicates the relative abundance of genera and metabolites. The colors of points display the different phyla of the microbiome. The circle represents the fecal microbiome, the square represents the oral microbiome, and the diamond represents plasma metabolites. CPR0 confirmed patients recover at discharge, CPR1 confirmed patients recover 1 year, HC healthy control, OTUs operational taxonomy units

In the process of occurrence, development and recovery in various diseases, there is a close relationship between the human microbiome and metabolites[23]. Thus, the correlations among the oral and gut microbiomes and metabonomics were analyzed in the gradual recovery process from CPR0 to CPR1 to HC. We included microbiomes and metabolites that showed an increasing or decreasing trend during the recovery process for Spearman’s correlation analysis. The results showed correlations among 26 fecal microbial OTUs, 28 oral microbial OTUs and 37 metabolites (Fig. 5, Additional file 2: Table S29). S1P was positively correlated with *Faecalibacterium*, *Bifidobacterium* and *Lachnospira* in the faeces and *Peptostreptococcus* and *Streptococcus* in the tongue coating. In addition, S1P was negatively correlated with *Burkholderiales* in the tongue coating. These results indicated that the beneficial microbiome and metabolites (such as S1P, *Bifidobacterium* and *Lachnospira*) gradually increased in the process of COVID-19 recovery, while the harmful microbiome and metabolites (such as *Burkholderiales*) gradually decreased.

Additionally, we conducted Spearman’s correlation analysis among clinical indicators, microbiome and metabolites in CPR1s and HCs (Additional file 2: Table S30, Additional file 3: Fig. S8). Lymphocytes were positively correlated with 2 fecal microbial OTUs, OTU1271 (*Subdoligranulum*) and OTU23 (*Erysipelotrichaceae*_*UCG-003*), and negatively correlated with 4-hydroxy-5-(phenyl)-valeric acid-O-sulfate. WBCs were positively correlated with oral microbial OTU115 (*Prevotella*) and negatively correlated with oral microbial OTU32 (*Atopobium*) and OTU60 (*Butyrivibrio*). Interestingly, alanine aminotransferase (ALT) was negatively correlated with Veillonella in the oral microbiome. Altogether, these results showed that alterations in the oral and gut microbiomes and metabonomics were closely related to the recovery of COVID-19.

## Discussion

COVID-19 has caused serious disease burden globally. Most COVID-19 patients experience a long recovery period after nucleic acid tests become negative. However, few studies have evaluated the features of the oral and gut microbiomes and metabonomics of these convalescents. Therefore, this study was the first to report the characteristics of the oral and gut microbiomes and metabonomics in one-year convalescents. Then, we clarified the dynamic alterations in the microbiome and metabonomics during the gradual recovery of COVID-19. Finally, we established prediction models based on the microbiome and metabolites of the patients at the time of discharge to predict the level of neutralizing antibodies one year later.

### Key metabolic and microbial biomarkers promote the recovery

In the context of 3PM, accurate prediction and treatment based on key biomarkers are particularly important [24, 25]. S1P, which is a signaling molecule, exerts multiple functions, including regulating the cytokine storm through its specific G protein-coupled receptors. S1P was significantly reduced in the peripheral blood of patients with COVID-19, and lower S1P meant worse prognosis [15, 22, 26]. In this study, S1P was found to have the lowest level at the time of discharge and gradually increased during the recovery process. SARS-CoV-2 infection led to an increase in proinflammatory cytokines and promoted an increase in S1P in the interstitial fluid, which in turn enhanced the secretion of cytokines in different cells, leading to a cytokine storm [27]. Anemia caused by cytokine storms reduces the production of S1P [28]. Additionally, the acute phase reaction in the liver led to a decrease in negative acute-phase proteins, including albumin and apoM, which acted as transporters to move S1P into the blood circulation [29, 30]. In addition, S1P can be used as a potential modulator and therapeutic target for SARS-CoV-2 infection [31, 32]. Moreover, in the process of disease recovery, there was a gradual increase in beneficial microbes and a decrease in harmful microbes [33, 34]. In this study, increasing S1P was positively correlated with the abundance of some beneficial microbes and negatively correlated with some harmful microbes. In the recovery process, the well-known probiotic *Bifidobacterium* gradually increased, and butyrate-producing *Faecalibacterium* also gradually increased in the gut microbiome. In contrast, lipopolysaccharide (LPS)-producing *Burkholderiales* gradually decreased in the oral microbiome. The increase in butyrate and *Bifidobacterium* and decrease in LPS could accelerate the recovery of many diseases [35–37]. Therefore, the increasing abundance of beneficial microbiomes and metabolites and the gradual decrease in harmful microbiomes benefited affected individuals and healthcare and promoted the recovery of COVID-19.

### Strengths and limitations in this study

This study has several strengths. We first reported the characterization of the oral and gut microbiome and metabonomics in a one-year convalescent of COVID-19. The previous study only reported six-month follow-up of gut microbiota richness in patients with COVID-19 [38]. The particularly striking finding was those compared with patients at the time of discharge, the oral and gut microbiome and metabolomics characteristics of the one-year convalescents were restored to a large extent but did not completely return to normal. In addition, the predictive models established based on the oral and gut microbiome and metabolomics of the confirmed patients at the time of discharge reached high efficiency in predicting their neutralizing antibody levels one year later. Moreover, all convalescents and healthy controls were from the same region with similar eating habits. Collectively, the results of this study are relatively rigorous. This study has some limitations. The prediction model for CPR1 neutralizing antibodies based on microbiome and metabolites is the result of a small sample size. Although the predictive models reached high efficiency, the small sample size may impact the robustness of this model. Large sample verification was needed before clinical practice. Moreover, it is an observational study and cannot indicate how the key microbiome and metabolites promote the recovery process. Future mechanistic studies are warranted to confirm the impact of the key microbiome and metabolites on the convalescents. It is also not certain whether similar alterations are observed in convalescents in other geographical regions.

## Conclusions

In conclusion, this study reported for the first time the characterization of the oral and gut microbiome and metabonomics in the one-year convalescents of COVID-19. The key microbiome and metabolites in the process of recovery were identified and provided new treatment ideas for accelerating recovery. And the predictive models based on the microbiome and metabolomics afford new insights for predicting the recovery situation which benefited affected individuals and the healthcare in the context of 3PM approach.

## Supplementary Information

**Additional file 1:** Consent informed and Supplementary methods

**Additional file 2: Table S1.** Clinical characteristics of participants in this study.** Table S2.** Level of neutralizing antibodies and IgG in the process of recovery.** Table S3.** Detail of the oral microbial α diversity index among the three groups.** Table S4.** Abundance and average composition at the genus level and phylum level of oral microbiome in each sample among the three groups.** Table S5.** Corresponding LDA value and P-value of the biomarkers of oral microbiome among the three groups.** Table S6.** Different degree of genus and phylum level (*P*-value) of oral microbiome in each sample among the three groups.** Table S7.** One year later neutralizing antibody and IgG of CPR0-L were significantly lower than those of CPR0-H.** Table S8.** Detail of the oral microbial α diversity index between CPR0-L and CPR0-H.** Table S9.** Different degree of genus and phylum level (*P*-value) of oral microbiome in each sample between CPR0-L and CPR0-H.** Table S10.** By random forest classifier model, the corresponding output value of each optimal oral microbial marker and the corresponding POD value for each sample in CPR0-L and CPR0-H.** Table S11.** Detail of the gut microbial α diversity index among the three groups.** Table S12.** Corresponding LDA value and* P*-value of the biomarkers of gut microbiome among the three groups.** Table S13.** Different degree of genus and phylum level (*P*-value) of gut microbiome in each sample among the three groups.** Table S14.** Relative abundance and distribution of the key gut microbial OTUs between CPR1 and HC groups.** Table S15.** Detail of the gut microbial α diversity index between CPR0-L and CPR0-H.** Table S16.** Different degree of genus and family level (P-value) of gut microbiome in each sample between CPR0-L and CPR0-H.** Table S17.** By random forest classifier model, the corresponding output value of each optimal gut microbial marker and the corresponding POD value for each sample in CPR0-L and CPR0-H.** Table S18.** Raw data obtained from positive ion mode of plasma metabonomics among the three groups.** Table S19.** Raw data obtained from negative ion mode of plasma metabonomics among the three groups.** Table S20.** Abundance and average composition of plasma metabonomics among the three groups.** Table S21.** Different degree of family level (*P*-value) of plasma metabonomics among the three groups.** Table S22.** Correlation between 204 different metabolites from CPR1s and CPR0s.** Table S23.** Correlation between 216 different metabolites from CPR1s and HCs.** Table S24.** Raw data obtained from positive ion mode of plasma metabonomics between CPR0-L and CPR0-H.** Table S25.** Raw data obtained from negative ion mode of plasma metabonomics between CPR0-L and CPR0-H.** Table S26.** Abundance and average composition of plasma metabonomics between CPR0-L and CPR0-H.** Table S27.** Different degree of family level (*P*-value) of plasma metabonomics between CPR0-L and CPR0-H.** Table S28.** By random forest classifier model, the corresponding output value of each optimal metabolites’ marker and the corresponding POD value for each sample in CPR0-L and CPR0-H.** Table S29.** Correlation in the gradual recovery process from CPR0 to CPR1 to HC.** Table S30.** Correlation in gut and oral microbiome and plasma metabonomics and clinical indicators between CPR1 and HC.

**Additional file 3: Fig. S1.** Gradual recovery of the oral microbiome.** Fig. S2.** Noninvasive prediction model for CPR1 neutralizing antibodies based on the oral microbiome.** Fig. S3.** Gradual recovery of the gut microbiome.** Fig. S4.** Noninvasive prediction model for CPR1 neutralizing antibodies based on the gut microbiome.** Fig. S5.** Correlation between 204 different metabolites from CPR1 and CPR0 was identified.** Fig. S6.** Correlation between 216 different metabolites from CPR1 and HC was figured out.** Fig. S7.** Noninvasive prediction model for CPR1 neutralizing antibodies based on plasma metabolomics.** Fig. S8.** Correlation in gut and oral microbiome and plasma metabonomics and clinical indicators between CPR1 and HC.

## Data Availability

The raw Illumina read data for all samples were available through the European Nucleotide Archive at the European Bioinformatics Institute under accession number PRJNA756623.

## Abbreviations

COVID-19: Coronavirus disease 2019
CPR0: Confirmed patients recover at discharge
CPR1: Confirmed patients recover 1 year
HC: Healthy control
CPR0-H: Confirmed patients who recovered at discharge with higher neutralizing antibodies 1 year later
CPR0-L: Confirmed patients who recovered at discharge with lower neutralizing antibodies 1 year later
PCA: Principal component analysis
PCoA: Principal coordinate analysis
PLS-DA: Partial least squares discrimination analysis
OPLS-DA: Orthogonal partial least squares discrimination analysis
NMDS: Nonmetric multidimensional scaling
LC–MS: Liquid chromatography-mass spectrometry
LDA: Linear discriminant analysis
POD: Probability of disease
CI: Confidence interval
AUC: Area under the curve
3PM: Predictive, preventive and personalized medicine
SARS-CoV-2: Severe acute respiratory syndrome coronavirus 2
OTU: Operational taxonomy unit
