# Supplementary material for: Characterization of oral and gut microbiome and plasma metabolomics in COVID-19 patients after 1-year follow-up
Source: Mil Med Res. 2022 Jun 17;9:32. doi: 10.1186/s40779-022-00387-y (PMC9204369; doi:10.1186/s40779-022-00387-y)
Supplement: Supplementary file 1 — Additional file 1: Consent informed and Supplementary methods [file 40779_2022_387_MOESM1_ESM.pdf]

Consent informed

Informed consent form for scientific research

(Translated from Chinese)

Dear participants,

We are from Department of Infectious Diseases, the First Affiliated Hospital of Zhengzhou University. We will free of charge help you monitor your healthy condition and record your clinical information and healthy/disease status or disease progression process. The collected tongue coating, fecal, and plasma samples from participants in hospital will be used for scientific research. These results and data from the hospital electronic medical records will provide auxiliary data for clinical diagnosis and treatment, and will be used for scientific research. Thank you for your corporation.

Number:	Diagnosis:

The information that we collect from this research project will be kept confidential. Information about you that will be collected during the research will be put away and no-one but the researchers will be able to see it. Any information about you will have a number on it instead of your name. Only the researchers will know what your number is and we will lock that information up with a lock and key. It will not be shared with or given to anyone except our research team.

The knowledge that we get from doing this research will be shared with you through community meetings before it is made widely available to the public. Confidential information will not be shared. There will be small meetings in the community and these will be announced. After these meetings, we will publish the results in order that other interested people may learn from our research.

I have read the foregoing information, or it has been read to me. I have had the opportunity to ask questions about it and any questions that I have asked have been answered to my satisfaction. I consent voluntarily to participate as a participant in this research.

Print Name of Participant__________________

Signature of Participant ___________________

Date ___________________________

Day/month/year

A literate witness must sign (if possible, this person should be selected by the participant and should have no connection to the research team). Participants who are illiterate should include their thumb-print as well.

I have witnessed the accurate reading of the consent form to the potential participant, and the individual has had the opportunity to ask questions. I confirm that the individual has given consent freely.

Print name of witness____________________ AND

Thumb print of participant

Signature of witness _____________________

Date _______________________

Day/month/year

Statement by the researcher/person taking consent

I have accurately read out the information sheet to the potential participant, and to the best of my ability made sure that the participant understands that the following will be done:

1. We will free of charge help you monitor your healthy condition and record your clinical information and healthy/disease status or disease progression process.

2. These data from hospital electronic medical records will be used for scientific research.

3. The collected tongue coating, fecal, and serum samples will be used for scientific research.

I confirm that the participant was given an opportunity to ask questions about the study, and all the questions asked by the participant have been answered correctly and to the best of my ability. I confirm that the individual has not been coerced into giving consent, and the consent has been given freely and voluntarily.

A copy of this ICF has been provided to the participant.

Print Name of Researcher/person taking the consent________________________

Signature of Researcher /person taking the consent__________________________

Date ___________________________

Day/month/year

Supplementary methods

Enrollment process

The 35 confirmed patients enrolled were diagnosed with a positive PT-PCR nucleic acid test on throat swab specimens a year ago. CPs' Nucleic acid of CPs turns negative after treatment. Convalescent patients were transferred to another independent isolated region under observation for 15 d. If all nucleic acid tests were negative during the isolation period, the convalescent patients could leave the isolation area. The convalescent patients provided samples 2 d before discharge. The convalescent patient provided stool, tongue coating and plasma samples at the time of 1-year after leaving the isolation area.

Inclusion and exclusion criteria

Nucleic acid test results were all positive when the convalescent patients were admitted 1-year ago. The inclusion criteria for healthy volunteers were similar to our previous studies[1]. Healthy controls who had the following diseases were excluded: coeliac disease, nonalcoholic fatty liver disease, irritable bowel syndrome (IBD), diabetes, metabolic syndrome and hypertension and oral disease. All participants who received antibiotics and/or probiotics within 8 weeks before providing samples were also excluded. All participants who have received the COVID-19 vaccine were excluded.

Tongue-coating collection

Each participant provided a tongue-coating sample from 7 am to 9 am. Tongue-coating samples were collected as our previous study [1]. On the day of sampling, the participants were asked to eat and brush their teeth after providing tongue-coating

samples. The participants rinsed their mouths twice with sterile water before taking the tongue-coating samples. A professional operator used a pharyngeal swab to scrape the posterior middle to the anterior middle region of the tongue-coating.

DNA extraction

Tongue-coating and faecal microbial DNA was extracted by the Qiagen Mini Kit (Qiagen, Hilden, Germany) as described previously [2]. The samples were processed by phenol trichloromethane DNA extraction using a bead beater to mechanically disrupt cells, followed by phenol-chloroform extraction. Then, we purified the DNA according to the manufacturer's instructions. The DNAs were quantified by the Qubit 2.0 Fluorometer (Invitrogen, Carlsbad, CA, USA), and molecular size was estimated using agarose gel electrophoresis. All microbial DNAs were diluted to 10 ng/μL for microbial analysis.

PCR amplification

The details of PCR amplification were performed according to our previous study [1]. PCR primers incorporated sample-specific barcodes for multiplex sequencing using the Illumina MiSeq System (paired-end 250-nt reads). Amplify the extracted DNA samples using primers (5’-ACTCCTACGGGAGGCAGCA-3’ and 5’-GGACTACHVGGGTWTCTAAT-3’) targeting the hypervariable V3-V5 region of the 16S rRNA gene. The PCR reaction system contained 10 ng of template DNA, 0.4 μl of Forward primer (5 μmol/L), 0.4 μl of Reverse primer (5 μμmol/L), 0.4 μl of Fast Pfu polymerase,4 μl of 5 × Fast Pfu buffer and 2 μl of 2.5 mmol/L dNTP (TransGen Biotech, Beijing, China). Perform four PCR reactions on each sample in a PCR machine (ABI

GeneAmp 9700) as follows: 95℃ for 2 min, 95℃ for 30 s, 55℃ for 30 s, 72℃ for 30 s, repeating 30 cycles and finally at 72℃ for 5 min. Pool the PCR products from the same sample. Separate, extract and purify the PCR products by Agarose gel (Axygen Biosciences, Union City, CA). At last, we used a fluorescence assay kit (Quant-iT PicoGreen, Invitrogen) to quantify the products.

Sequence data process

The amplified reads were processed according to the following steps: (1) overlape pair end sequenced reads of each library by the FLASH v.1.2.10 software. (2) perform more specific quality control on overlapping reads generated by FLASH using the customization of each program: 1) No ambiguous bases (N) were allowed in reads; 2) The mismatch rate in the overlapping area is not allowed to exceed 0.05; 3) No mismatches were allowed in the primer and barcode region. (3) de-multiplex and assign reads into different samples based on barcodes. (4) the chimeric sequences were detected and removed by UCHIME version 4.2.40 (version microbiome util-r20110519, http://drive5.com/uchime/gold.fa) to match Operational Taxonomy Units (OTUs).

Microbial diversity and taxonomic analysis

We calculated the microbial diversity according to sampling OTUs analysis. The R program package ‘vegan’ was used for calculating Simpson index, Shannon index, Chao index, ACE index and Observed_otus. The R package (http://www.R-project.org/) was used for Principal coordinate analysis (PCoA) to visualize interactions among bacterial communities according to OTUs distribution [3]. We used the Phyloseq package to calculate the weighted and unweighted unifrac distances. PCoA was

conducted based on unweighted and weighted uniFrac. A heatmap for the key variables was constructed by the Heatmap Builder according to the *P* value < 0.05. The specific characterization of microbiota to distinguish taxonomic types was analyzed by linear discriminant analysis (LDA) effect size (LEfSe) method (http://huttenhower.sph.harvard.edu/lefse/). LEfSE was applied to infer differential abundance of taxa between each group. The significantly different taxa were identified by LEfSe on the Kruskal-Wallis rank sum test with the LDA score (log10) =3 or 3.6[4].

Construction of POD

A fivefold cross-validation was conducted on a random forest model by the abundance profile of the optimal OTUs markers (R 3.4.1, randomForest 4.6 - 12 package). The cross-validation error curve was calculated through five trials of the fivefold cross validation. The cut-off point through the minimum error plus the standard deviation (SD) was defined as the point with the minimum cross-validation error. The optimal OTUs was defined by the smallest number of OTUs sets with the error less than the cut-off value. In addition, the POD index was calculated by the optimal set of OTUs. We used the receiver operating characteristic (ROC) curve to evaluate the model through the R package pROC. The AUC value indicated the the ROC effect.

Untargeted metabolomics detection

Untargeted metabolomics testing on liquid chromatography-mass spectrometry (LC-MS) analysis was carried out on all plasma samples. We slowly thawed all plasma samples at 4℃ and mixed 100 μl ofplasma with 400 μl pre-cooled methanol. Then, we centrifugated the mixed liquor at 12,000 r/min and 4℃ for 10 min and extracted the

supernatant and blow-dried it by vacuum concentration. In addition, we dissolved the dried samples with 150 μl of 2-chlorobenzylamine (4×10^{-6}) methanol aqueous solution (4:1, 4°C). Furthermore, we filtered the supernatant through a 0.22 μm membrane for LC-MS analysis. At the same time, quality control (QC) samples were prepared. The liquid chromatographic separation was performed on a Thermo Ultimate 3000 system (Thermo Fisher Scientific Inc., Waltham, MA, United States) equipped with a Waters ACQUITY UPLC® HSS T3 column (150 × 2.1 mm, 1.8 μm). We adjusted the flow rate at 0.25 ml/min and maintained the column temperature at 40°C. The mobile phase was made up of 0.1% formic acid in water and 0.1% formic acid in acetonitrile or 5 mmol/L ammonium formate in water and acetonitrile. Injection of 2 μl of each sample was done after equilibration. The MS experiment was conducted on the Thermo Q Exactive Focus mass spectrometer (Thermo Fisher Scientific Inc., Waltham, MA, United States) with the spray voltage of 3.8 kV and −2.5 kV in positive ion mode (ESI^+) and negative ion mode (ESI^-), respectively.

Metabolomics data preprocessing and annotation

The IS was used to evaluate the stability of the instrument. We calculated the minimum metabolic value for the specific samples whose metabolic level was lower than the quantitative lower limit. Then, we normalized the sum of all metabolic characteristics. Metabolic characteristics whose QC was greater than 30% relative standard deviation (RSD) were discarded. In order to select metabolite with significant differences, statistical analysis of $\log_{10}$-converted data was conducted after normalization and imputation. These metabolic characteristics were identified by precise mass

spectrometry. We performed the multivariate data analysis, including Pareto-scaled principal component analysis (PCA), partial least-squares discriminant analysis (PLS-DA) and orthogonal partial least-squares discriminant analysis (OPLS-DA).

Immunoassay of SARS-CoV-2 neutralizing antibody and IgG

Levels of neutralizing antibody against SARS-CoV-2 in serum were tested through the ELISA using kits (GenScript Biotech., Ltd., Nanjing, China) [5]. The neutralize reactions were performed according to the following steps: (1) In separate tubes, mix the diluted positive control, diluted negative control, and the samples with the diluted HRP-RBD solution with a volume ratio of 1:1. For example, mix 60 µl positive control with 60 µl HRP-RBD solution. Incubate the mixtures at 37℃ for 30 min. (2) Add 100 µl each of the positive control mixture, the negative control mixture, and the sample mixture to the corresponding wells. (3) Cover the plate with plate sealer and incubate at 37℃ for 15 min. (4) Remove the plate sealer and wash the plate with 260 µl of 1 × wash solution for 4 times. (5) Pat the plate on paper towel to remove residual liquid in the wells after washing steps, substrate reaction and absorbance measurement. (6) Add 100 µl of TMB solution to each well and incubate the plate in the dark at 20 − 25℃ for 15 min (start timing after the addition of TMB solution to the first well). (7) Add 50 µl of stop solution to each well to quench the reaction. (8) Read the absorbance in the microtiter plate reader at 450 nm immediately. The absorbance of negative control was not allowed ≤ 1.0, and the absorbance of positive control was not allowed ≥ 0.3. The neutralizing antibody inhibition rate = (1 − OD value of sample/OD value of negative control) × 100%. The cutoff value of the kit was 30% (a rate ≥ 30% was defined as

positive, and a rate < 30% was defined as negative).

Levels of IgG against SARS-CoV-2 in serum were tested through the direct chemiluminometric microparticle technology using kits (YHLO Biotech Co., Ltd., Shenzhen, China) [6]. The iFlash 3000-C chemiluminescence immunoassay analyzer (Shenzhen YHLO Biotech Co., Ltd., China) was used for the test. The positive judgment value of the kit was 10 U/ml (a value > 10 U/ml was defined as positive, and a value < 10 U/ml was defined as negative). The IgG levels were calculated as $\log_{10}$ (value).

References

1. Ren Z, Wang H, Cui G, Lu H, Wang L, Luo H, et al. Alterations in the human oral and gut microbiomes and lipidomics in COVID-19. Gut. 2021;70(7):1253-65.
2. Ren Z, Li A, Jiang J, Zhou L, Yu Z, Lu H, et al. Gut microbiome analysis as a tool towards targeted non-invasive biomarkers for early hepatocellular carcinoma. Gut. 2019;68(6):1014-23.
3. McMurdie PJ, Holmes S. phyloseq: an R package for reproducible interactive analysis and graphics of microbiome census data. PLoS One. 2013;8(4):e61217.
4. Ling Z, Liu X, Jia X, Cheng Y, Luo Y, Yuan L, et al. Impacts of infection with different toxigenic Clostridium difficile strains on faecal microbiota in children. Sci Rep. 2014;4:7485.
5. Tan CW, Chia WN, Qin X, Liu P, Chen MI, Tiu C, et al. A SARS-CoV-2 surrogate virus neutralization test based on antibody-mediated blockage of ACE2-spike protein-

protein interaction. Nat Biotechnol. 2020;38(9):1073-8.

6. Qian C, Zhou M, Cheng F, Lin X, Gong Y, Xie X, et al. Development and multicenter performance evaluation of fully automated SARS-CoV-2 IgM and IgG immunoassays. Clin Chem Lab Med. 2020;58(9):1601-7.
