# Supplementary material for: Characterization of oral and gut microbiome and plasma metabolomics in COVID-19 patients after 1-year follow-up
Source: Mil Med Res. 2022 Jun 17;9:32. doi: 10.1186/s40779-022-00387-y (PMC9204369; doi:10.1186/s40779-022-00387-y)
Supplement: Supplementary file 3 — Additional file 3: Fig. S1. Gradual recovery of the oral microbiome. Fig. S2. Noninvasive prediction model for CPR1 neutralizing antibodies based on the oral microbiome. Fig. S3. Gradual recovery of the gut microbiome. Fig. S4. Noninvasive prediction model for CPR1 neutralizing antibodies based on the gut microbiome. Fig. S5. Correlation between 204 different metabolites from CPR1 and CPR0 was identified. Fig. S6. Correlation between 216 different metabolites from CPR1 and HC was figured out. Fig. S7. Noninvasive prediction model for CPR1 neutralizing antibodies based on plasma metabolomics. Fig. S8. Correlation in gut and oral microbiome and plasma metabonomics and clinical indicators between CPR1 and HC. [file 40779_2022_387_MOESM3_ESM.pdf]

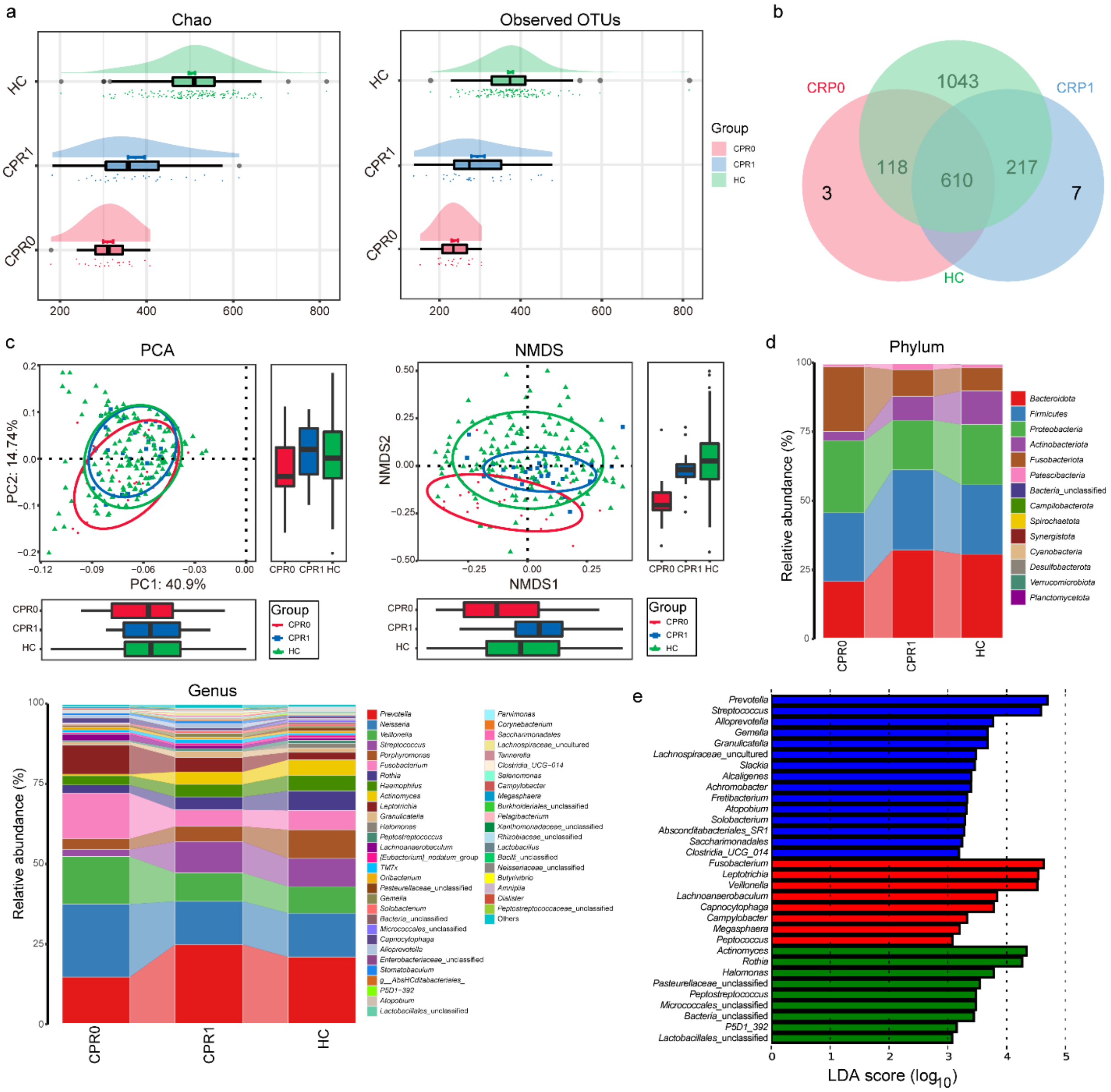

Fig. S1 Gradual recovery of the oral microbiome. a As estimated by Chao index and Observed OTUs, the oral microbial diversity gradually recovered. **b** Veen diagram displayed the shared and different OTUs between the three groups. **c** PCA and NMDS analysis showed that the compositions and diversity gradually recovered. **d** Average compositions and relative abundance of the microbial community in three groups at the phylum and genus levels. **e** Oral microbial markers were identified at LDA score (log_{10}) > 3 among the three groups. CPR0 confirmed patients recover at discharge, CPR1 confirmed patients recover 1 year, HC healthy control, OTUs operational taxonomy units, PCA principal component analysis; NMDS nonmetric multidimensional scaling, LDA linear discriminant analysis

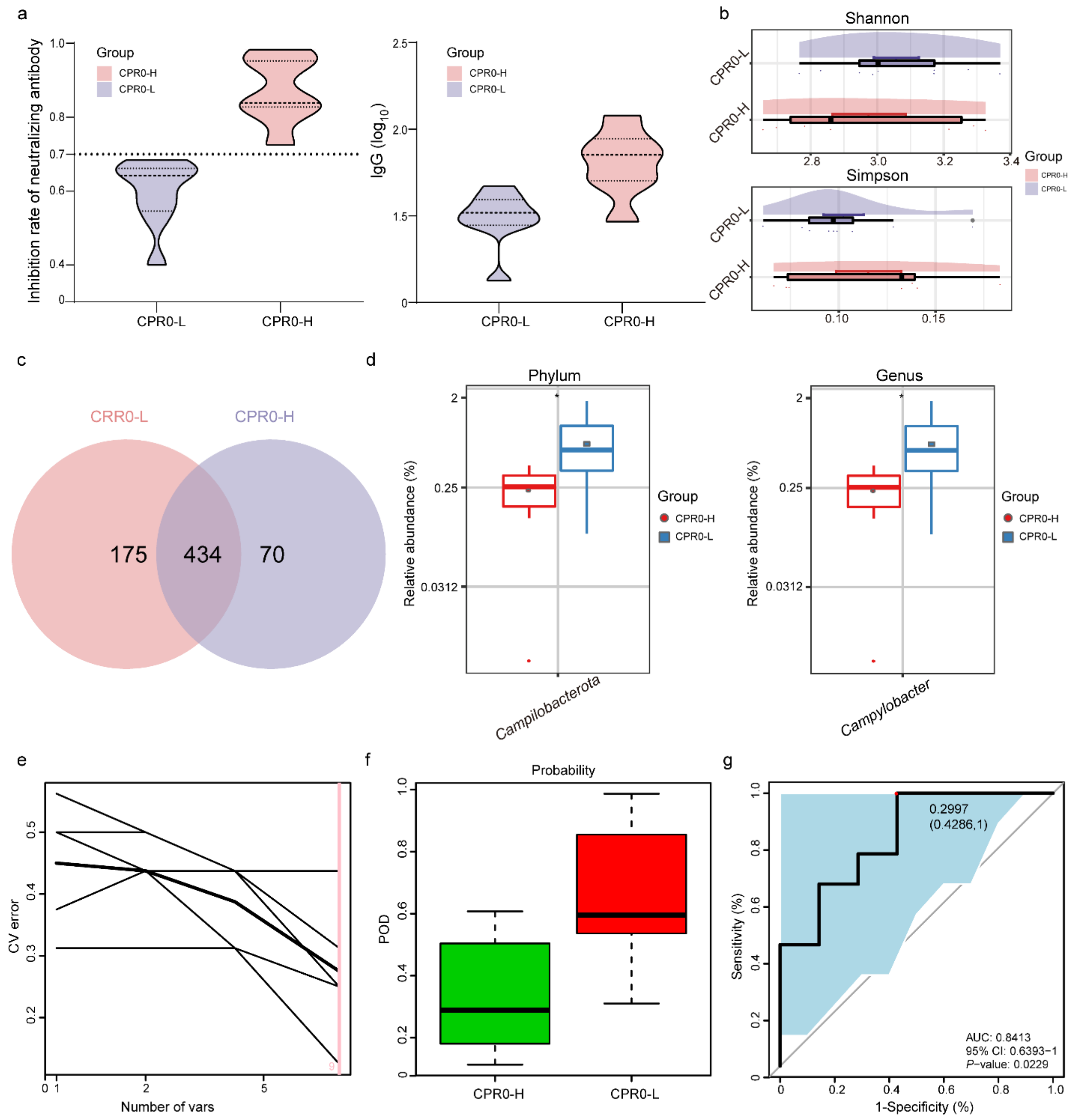

Fig. S2 Noninvasive prediction model for CPR1 neutralizing antibodies based on the oral microbiome. a One year later, the neutralizing antibody and IgG of CPR0-L were significantly lower than those of CPR0-H. **b** As estimated by the Shannon index and Simpson index, there was no significant difference in the α diversity between CPR0-L and CPR0-H. **c** Venn diagram based on OTU distribution showed that the compositions of the two groups were similar. **d** The key oral microbiome with significant differences between CPR0-L group and CPR0-H group was identified at the phylum and genus levels. **e** Nine OTUs markers were selected as the optimal marker set by random forest models. **f** POD index was significantly increased in CPR0-L compared with CPR0-H. **g** POD index

achieved an AUC value of 0.8413 (95% CI 0.6393 - 1) between CPR0-L and CPR0-H. $^{*}P < 0.05$, comparison between CPR0-L group and CPR0-H group. CPR0-H confirmed patients who recovered at discharge with higher neutralizing antibodies 1 year later, CPR0-L confirmed patients who recovered at discharge with lower neutralizing antibodies 1 year later, OTUs operational taxonomy units, CV error cross-validation error, POD probability of disease, CI confidence interval, AUC area under the curve

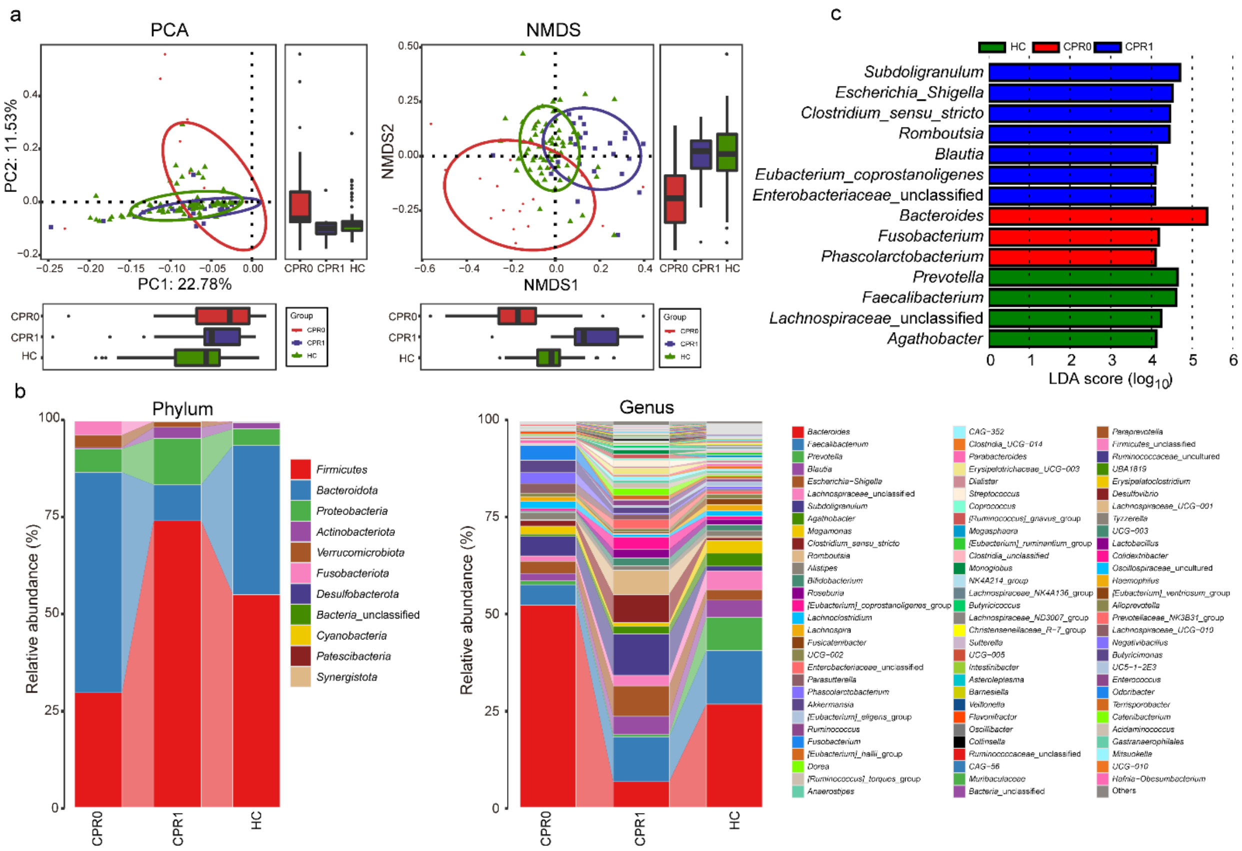

Fig. S3 Gradual recovery of the gut microbiome. a PCA and NMDS analysis showed that the compositions and diversity gradually recovered. **b** average compositions and relative abundance of the microbial community in three groups at the phylum and genus level. **c** Gut microbial markers were identified at LDA score (log_{10}) > 4 among the three groups. CPR0 confirmed patients recover at discharge, CPR1 confirmed patients recover 1 year, HC healthy control, OTUs operational taxonomy units, PCA principal component analysis, NMDS nonmetric multidimensional scaling, LDA linear discriminant analysis

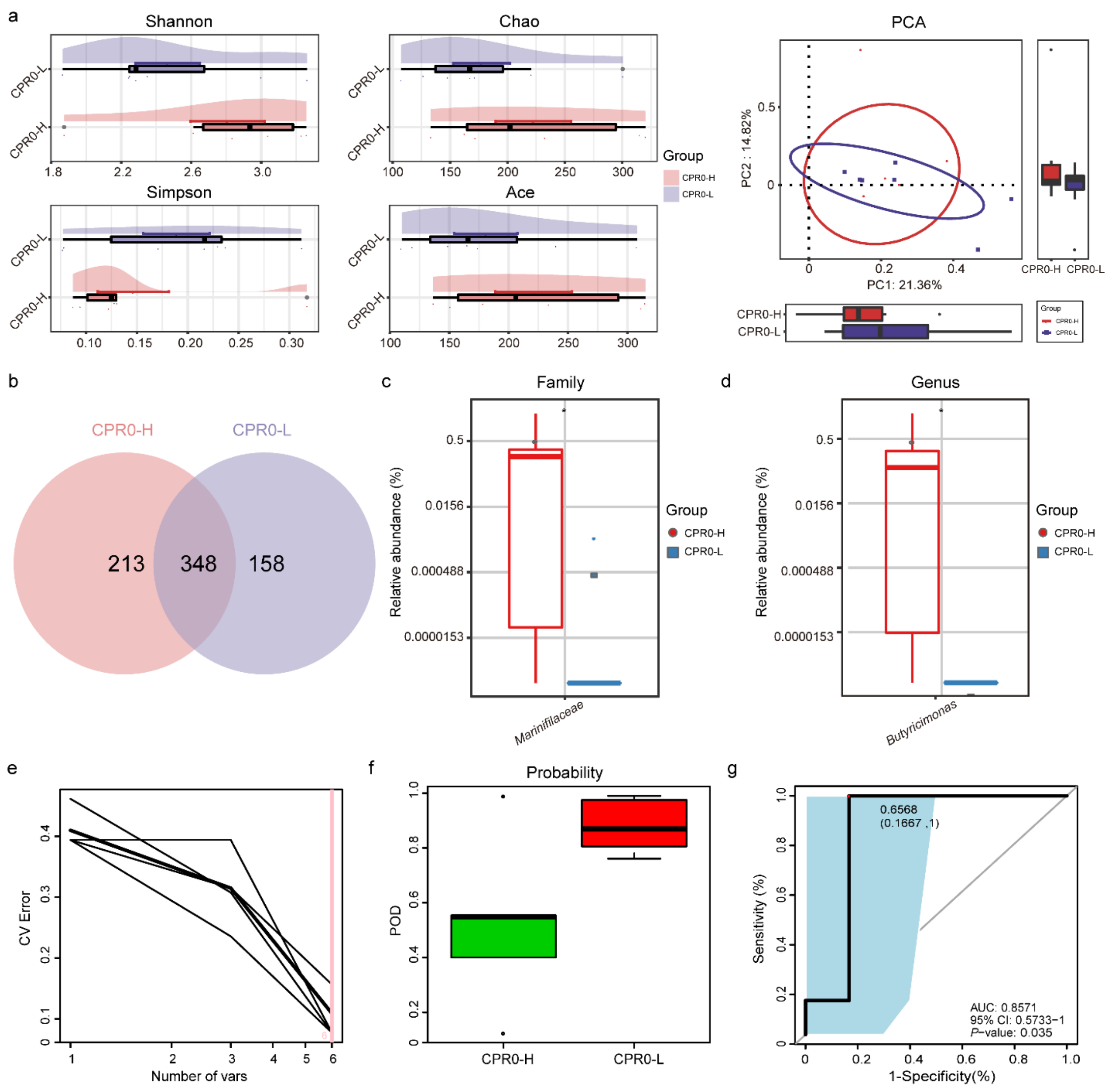

Fig. S4 Noninvasive prediction model for CPR1 neutralizing antibodies based on the gut microbiome. a As estimated by the Shannon index, Simpson index, Chao index, Ace index and PCA, there was no significant difference in the microbial diversity of the gut microbial community between CPR0-L and CPR0-H. **b** Venn diagram based on OTUs distribution showed that the compositions of the two groups were similar. **c** At the family level, *marinifilaceae* was significantly depleted in CPR0-L compared with CPR0-H. **d** *Butyricimonas,* which was significantly reduced in CPR0-L compared with CPR0-H at the genus level. **e** Six OTUs markers were selected as the optimal marker set by random forest models. **f** POD index was significantly increased in CPR0-L compared with CPR0-H. **g** POD index achieved an AUC value of 85.71% with a 95% CI of 57.33% to 100% between CPR0-L

and CPR0-H. The AUC curve showed a high prediction efficiency, confirming the ability of the gut microbiome to predict CPR1' neutralizing antibodies. $^{*}P < 0.05$, comparison between CPR0-L group and CPR0-H group. CPR0-H confirmed patients who recovered at discharge with higher neutralizing antibodies 1 year later, CPR0-L confirmed patients who recovered at discharge with lower neutralizing antibodies 1 year later, PCA principal component analysis, OTUs operational taxonomy units, CV error cross-validation error, POD probability of disease, CI confidence interval, AUC area under the curve

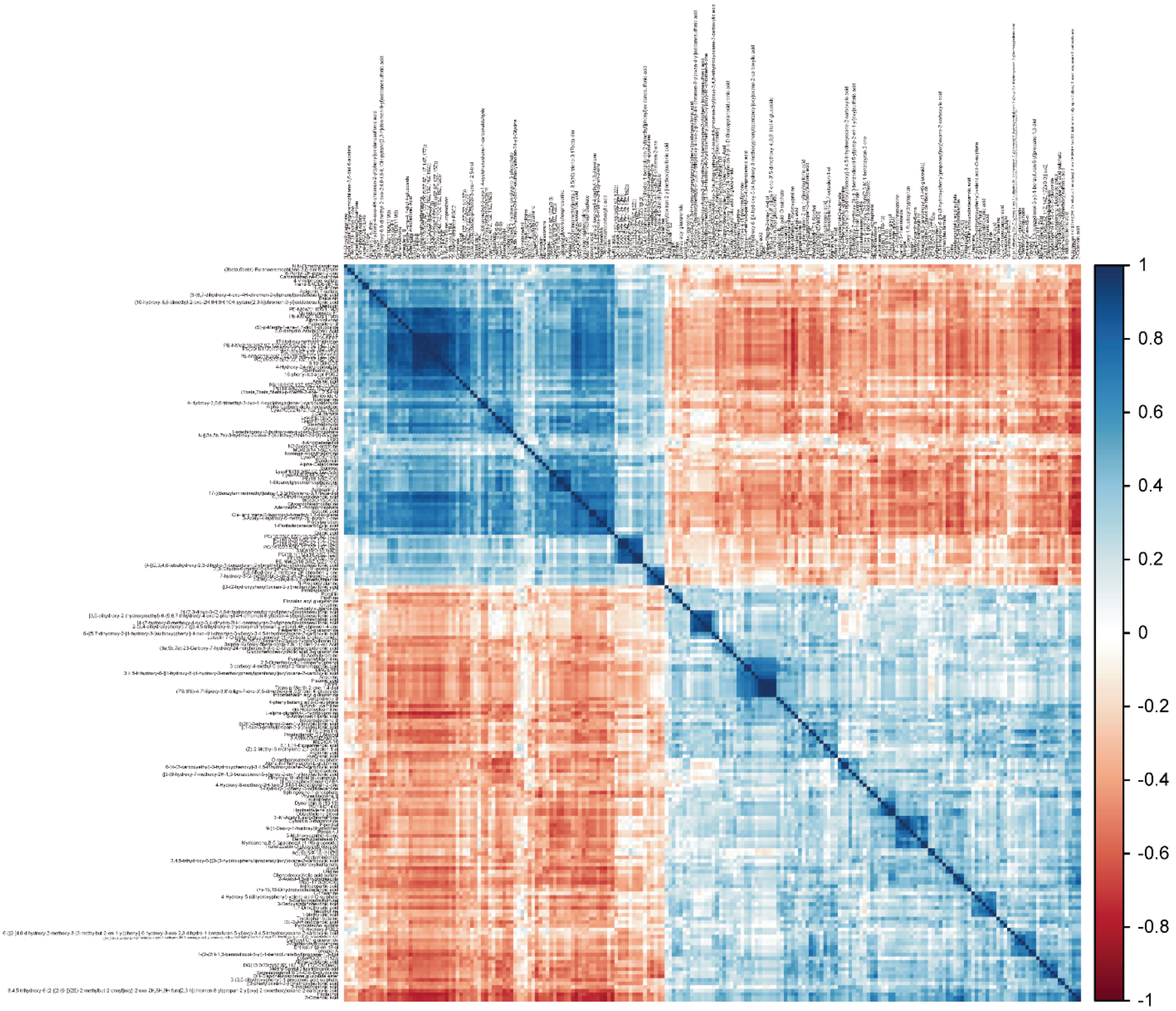

Fig. S5 Correlation between 204 different metabolites from CPR1 and CPR0 was identified

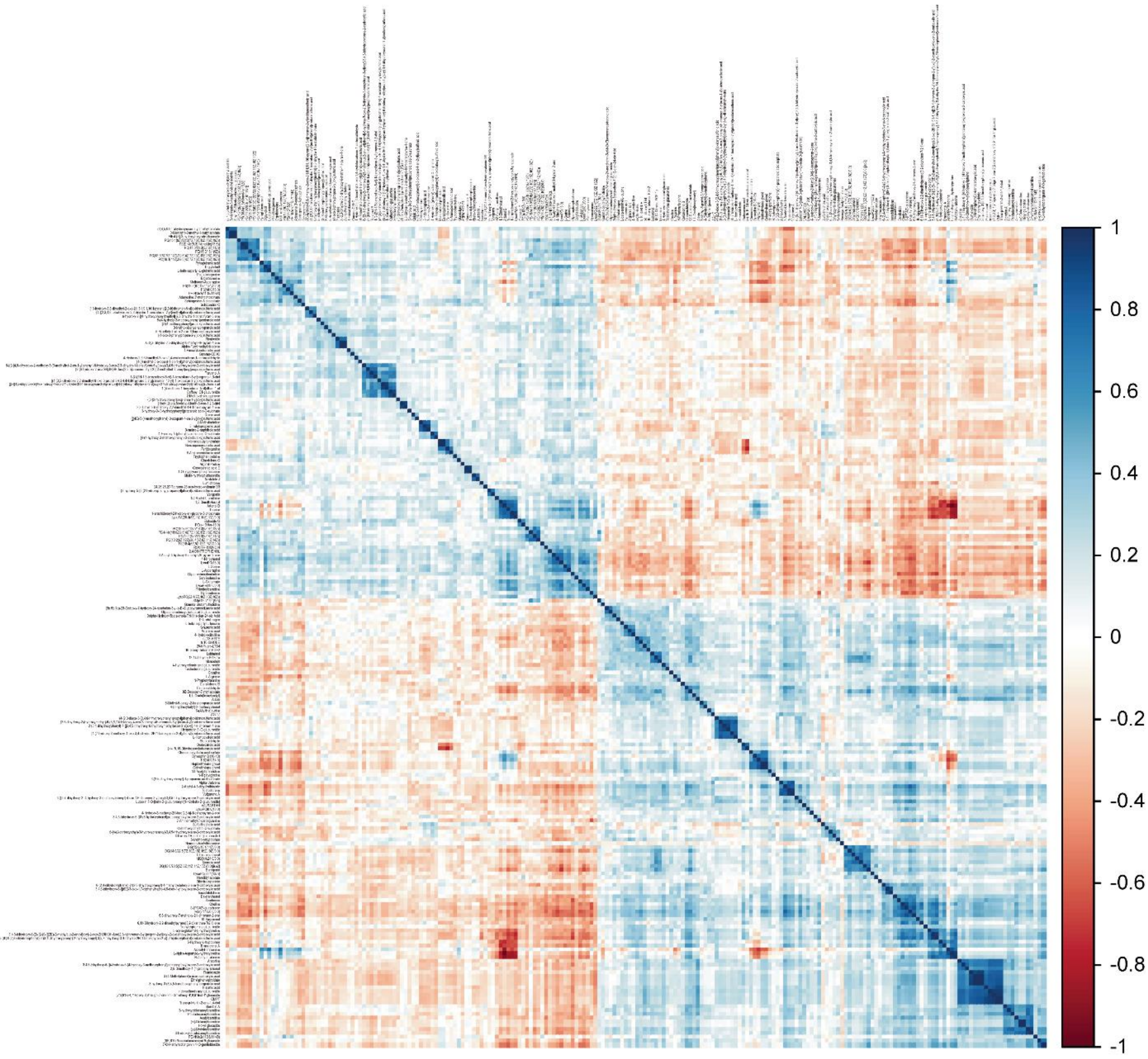

Fig. S6 Correlation between 216 different metabolites from CPR1 and HC was figured out

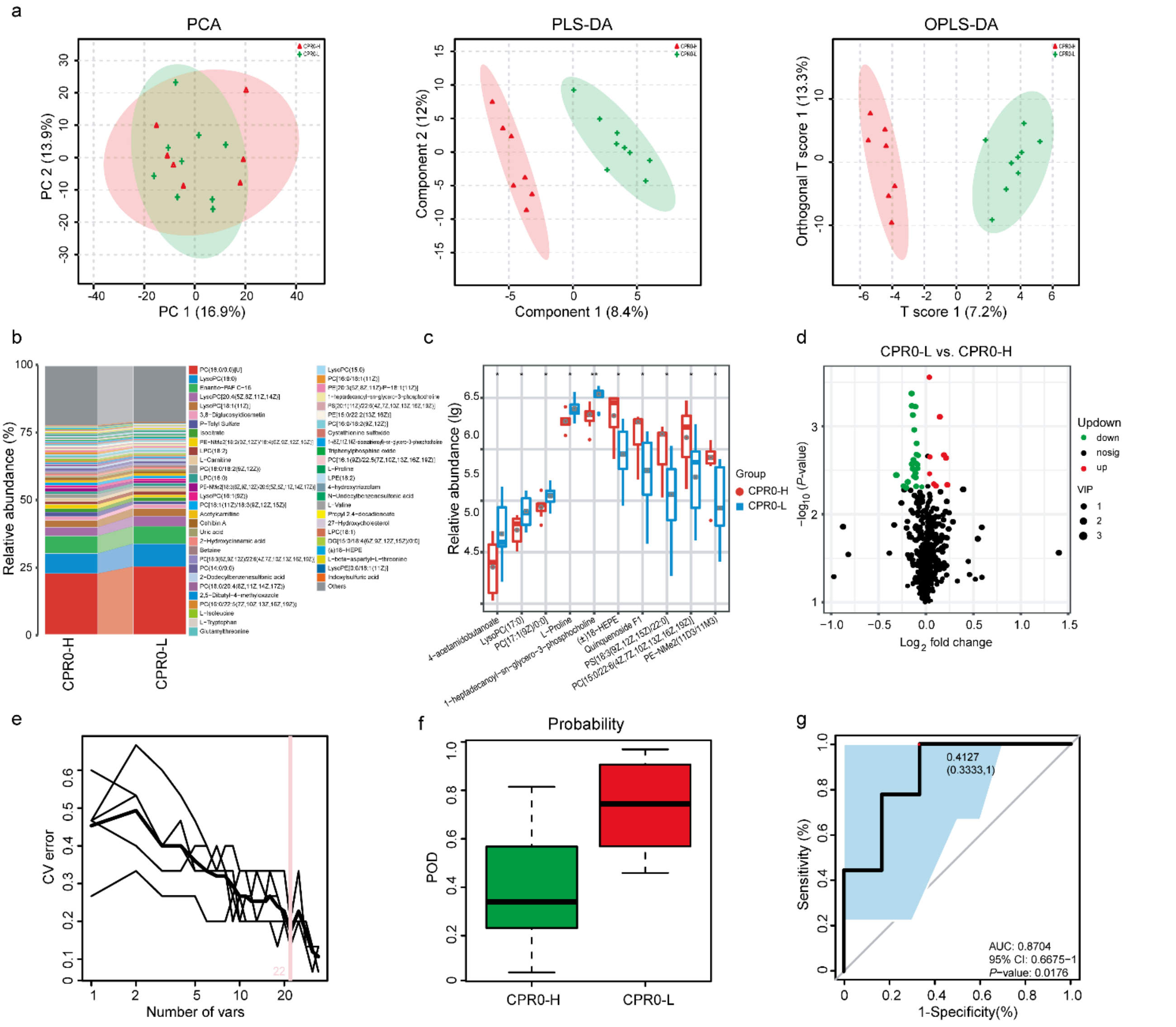

Fig. S7 Noninvasive prediction model for CPR1 neutralizing antibodies based on plasma metabolomics. a PCA, PLD-DA and OPLS-DA of the plasma metabonomics showed differences in composition between CPR0-L and CPR0-H. **b** Average compositions and relative abundance of the metabonomics community between CPR0-L and CPR0-H. **c** Abundance of 23 metabolites, including (±)18-HEPE and quinquenoside F1, was significantly decreased in CPR0-L. However, the abundance of 11 metabolites, including 4-acetamidobutanoate and LysoPC (17:0), was enriched in CPR0-L. **d** Annotated metabolites were analyzed and compared between CPR0-L and CPR0-H. **e** Twenty-two plasma metabolite markers were selected as the optimal marker set by random forest models. **f** POD index was significantly increased in CPR0-L compared with CPR0-H. **g** POD index achieved an AUC value of 0.8704 (95% CI 0.6675 - 1) between CPR0-L and CPR0-H. The AUC curve showed a high prediction efficiency, confirming the ability of plasma metabonomics to predict CPR1' neutralizing

antibodies. CPR0-H confirmed patients who recovered at discharge with higher neutralizing antibodies 1 year later, CPR0-L confirmed patients who recovered at discharge with lower neutralizing antibodies1 year later, PCA principal component analysis, PLS-DA partial least squares discrimination analysis, OPLS-DA orthogonal partial least squares discrimination analysis, Log_2FC log fold change, CV error cross-validation error, POD probability of disease, CI confidence interval, AUC area under the curve

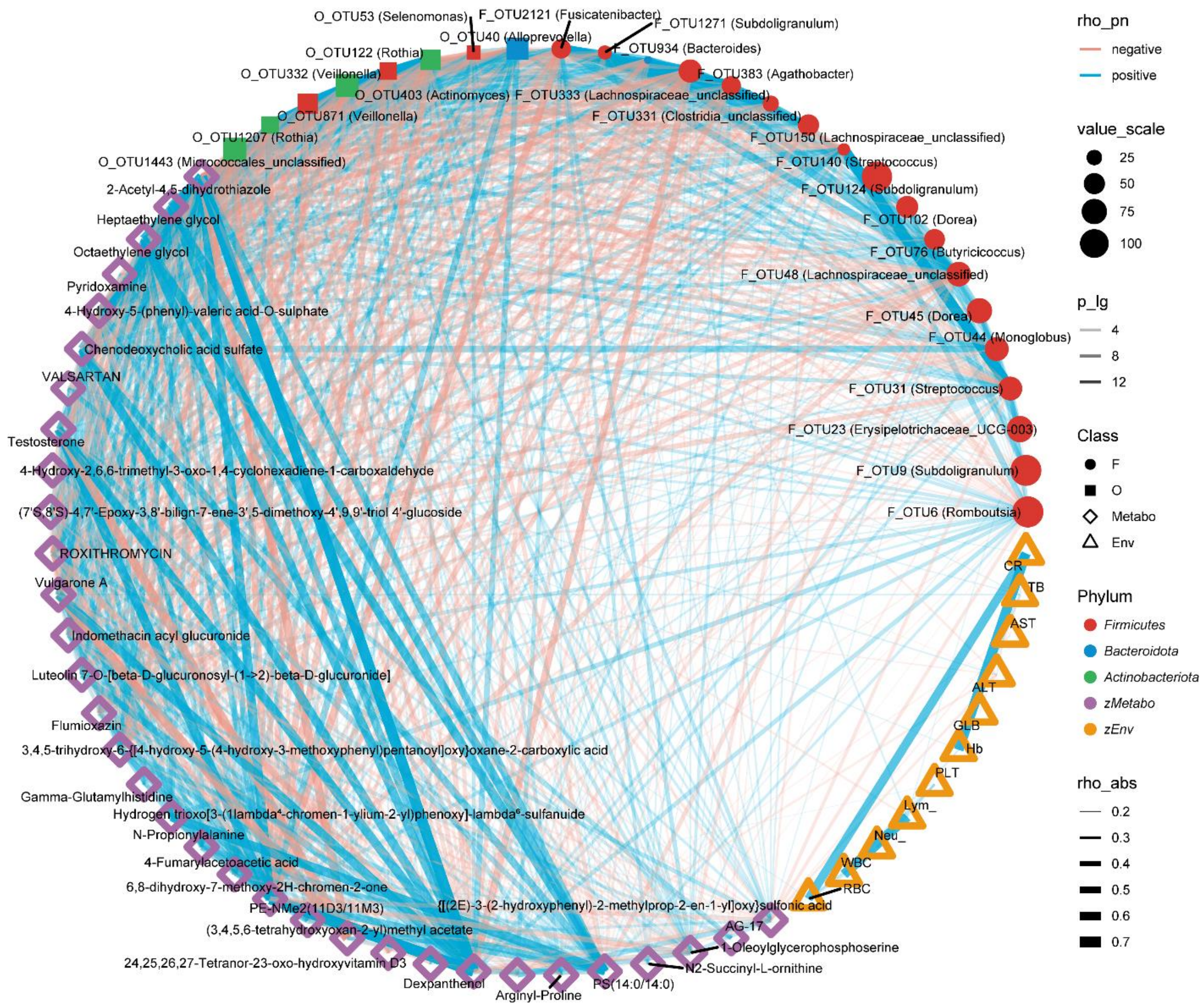

Fig. S8 Correlation in gut and oral microbiome and plasma metabonomics and clinical indicators between CPR1 and HC. The relationship among 18 oral microbial OTUs, 8 fecal microbial OTUs, 33 metabolites and 11 clinical indicators in CPR1s and HCs were identified. Red lines meant negative correlations, blue lines meant positive correlations, and the width of the lines represented the strength of the correlation (Spearman). The transparency of the lines represented the negative logarithm of the p-value of correlation, translucent lines meant (-lgP) > 5 and opaque lines meant (-lgP) >10. The size of the points indicated the relative abundance of genera and metabolites. The colors of points displayed the different phyla of the microbiome. The circle represented the fecal microbiome, the square represented the oral microbiome, the diamond represented plasma metabolites and the triangle represented clinical indicators.
